# Linking reinforcement learning, working memory, and choice dynamics to age and symptoms of anxiety and depression in adolescence

**DOI:** 10.1016/j.dcn.2025.101626

**Published:** 2025-10-08

**Authors:** Erik R. Frogner, Andreas Dahl, Rikka Kjelkenes, Torgeir Moberget, Anne G.E. Collins, Lars T. Westlye, Mads L. Pedersen

**Affiliations:** a Department of Psychology, University of Oslo, Oslo, Norway; b Center for Precision Psychiatry, Division of Mental Health and Addiction, Oslo University Hospital, Oslo, Norway; c Department of Psychology, Pedagogy and Law, School of Health Sciences, Kristiana University of Applied Sciences, Oslo, Norway; d Helen Wills Neuroscience Institute, University of California, Berkeley, United States; e Department of Psychology, University of California, Berkeley, United States; f KG Jebsen Centre for Neurodevelopmental Disorders, University of Oslo, Oslo, Norway

**Keywords:** Adolescence, Reinforcement learning, Working memory, Computational modeling, Anxiety, Depression

## Abstract

Adolescence is a sensitive period characterized by significant neurocognitive development, with important implications for learning and decision-making. Working memory and reinforcement learning are essential for decision making and real-life dynamic adaptation to the environment and are often affected in individuals with anxiety and depression. Using a cognitive computational approach, we investigated associations between working memory, reinforcement learning, age, and symptoms of generalized anxiety and depression in 193 adolescents aged 12–24 years. Participants completed the Reinforcement Learning Working Memory (RLWM) task. To gain insight into the dynamics underlying instrumental learning behavior, we employed a computational model that combines the RLWM model with a Linear Ballistic Accumulator (RLWM-LBA), to quantify processes related to working memory and reinforcement learning, as well as choice dynamics underlying reaction times. We observed an age-related increase in task performance, but no differences depending on symptoms. Bayesian regression models revealed strong evidence for an association between age and the start point variability parameter of the LBA module, suggesting reduced choice stochasticity in older adolescents. In line with the behavioral findings, we found anecdotal to moderate evidence for no associations between RLWM-LBA parameters and symptom sum scores. Lastly, we trained regression models testing the utility of RLWM-LBA parameters for predicting age and symptom burden, yielding poor predictive performance. This study highlights differences in choice dynamics underlying age-related improvements in instrumental learning, while finding that cognitive differences identified in case-control studies did not generalize to symptom variation in this sample from the general adolescent population.

## 1 Introduction

Adolescence is a transitional phase between childhood and adulthood, marked by rapid developmental changes in both the body and behavior. During this period, the brain undergoes numerous adaptive changes that prepare the individual for future life challenges, potentially leveraging a second window of brain plasticity (Larsen and Luna, 2018, Piekarski et al., 2017). Concurrently, adolescence is a critical period for the emergence of mood disorders (Paus et al., 2008, Solmi et al., 2022). Anxiety disorders and depression are among the most common mental disorders during adolescence (Kessler et al., 2012), and are highly comorbid (Kessler et al., 2005). Adolescent anxiety and depression have been associated with adverse future outcomes, including an elevated risk of recurrent episodes in adulthood (Copeland et al., 2009, Pine et al., 1998), suicidality (Asselmann et al., 2018, Goldman-Mellor et al., 2014), and unemployment in young adulthood (Rodwell et al., 2018). Also, sub-threshold symptoms of anxiety and depression during adolescence are associated with increased risk of subsequent psychiatric disorders in adulthood (Fergusson et al., 2005, Georgiades et al., 2006, Jinnin et al., 2016, Johnson et al., 2009, Noyes et al., 2022, Pine et al., 1999).

Cognitive dysfunction is common across psychiatric diagnoses (Abramovitch et al., 2021) including individuals with internalizing symptoms (Chavez-Baldini et al., 2023, Zhu et al., 2019), and often precede the onset of symptoms (Dickson et al., 2012, Gur et al., 2014). Computational psychiatry is a growing field with an overarching aim of identifying cognitive and behavioral mechanisms of mental disorders. This is done by applying and developing computational models and methods to understand the complex mechanisms underlying mental disorders and psychiatric symptoms (Huys et al., 2016, Huys et al., 2021). One of the most used classes of computational models are reinforcement learning models (Sutton and Barto, 2018). In the context of traditional learning theory, reinforcement learning is a theoretical and computational framework for understanding how agents learn from interactions with their environment. It combines principles of classical and operant conditioning, trial-and-error learning, feedback-driven reinforcement, and cognitive processes to model learning and decision-making. Reinforcement learning captures learning as an incremental process where the values assigned to choices are updated based on the difference between expected and observed reward, known as the reward prediction error, in proportion to a learning rate (Sutton and Barto, 2018). The learning rate reflects the extent to which new information (i.e. the reward prediction error) influences this updating of estimated choice values.

Anxiety and depression have previously been associated with disruptions in reinforcement learning processes (Bishop and Gagne, 2018, Halahakoon et al., 2020), although findings are varied. Some studies suggest that observed reinforcement learning disturbances may stem from increased learning from punishment (Aylward et al., 2019, Beevers et al., 2013), as reflected in higher learning rates for punishing outcomes compared to healthy individuals. Alternatively, disturbances may stem from reduced reward sensitivity (Auerbach et al., 2022, Huys et al., 2013), which refers to how reinforcing positive outcomes are for learning. By disproportionately learning from negative experiences, both could lead to a negative bias, a tendency to preferentially process negatively valenced information (Ironside et al., 2020, Pedersen et al., 2021), which is a commonly suggested candidate cognitive mechanism for the development and maintenance of anxiety and depressive symptoms (Beck, 2008, MacLeod et al., 1986). Recently, a computational simulation meta-analysis revealed that, compared to healthy peers, individuals with anxiety and/or depressive disorders exhibited elevated punishment learning rates and slightly lower reward learning rates (Pike and Robinson, 2022). Thus, differences in punishment learning rate may be linked to the promotion and maintenance of negative affective bias symptoms.

While considerable attention has been given to the role of reinforcement learning in anxiety and depression, there has been limited exploration of how other cognitive processes might contribute to the observed differences in reinforcement learning. The basal ganglia-based reinforcement learning processes have been shown to be supported by multiple other systems, including hippocampus dependent episodic memory (Bornstein and Daw, 2012, Bornstein and Norman, 2017, Davidow et al., 2016, Hong et al., 2024) and executive functions such as working memory (Collins and Frank, 2012). Working memory and reinforcement learning have been shown to be neurally and behaviorally intertwined, and interact in many learning contexts, even simple ones thought to rely on a single learning process such as reinforcement learning (Collins and Frank, 2012, Yoo and Collins, 2022). Working memory facilitates the immediate storage of information; however, the representations it holds are believed to decay over time, and the number of simultaneous representations is limited (Oberauer et al., 2018). In contrast, reinforcement learning operates through a gradual, incremental process of trial and error and is considered to have an unlimited capacity for storing learned information (Sutton and Barto, 2018). Thus, there is a trade-off between using the fast working memory system, where information is capacity-limited and decays over time, versus the slower, capacity-unlimited reinforcement learning system. In addition to disturbances in reinforcement learning, working memory deficits are commonly observed in anxiety and depression (Balderston et al., 2017; Le et al., 2017; Nikolin et al., 2021; Rose and Ebmeier, 2006; Schumacher et al., 2024). However, there is a paucity of research examining how deficits in working memory and reinforcement learning may interact and reciprocally influence one another in the context of anxiety and depression.

Behavioral testing combined with computational modeling can disentangle the simultaneous contributions of reinforcement learning and working memory. The Reinforcement Learning Working Memory (RLWM) task is a deterministic reward-learning task that taxes working memory by varying the number of stimulus-action associations (i.e. set sizes) to learn across blocks, and has been used, along with the RLWM computational model, to isolate contributions of working memory and reinforcement learning during learning (Collins, 2018, Collins and Frank, 2012, Collins and Frank, 2018). Participants typically use working memory for learning during low set sizes when the number of representations to hold are within capacity, and otherwise compensate with reinforcement learning. Furthermore, working memory appears to inform reward expectations used by the reinforcement learning system, especially during low set sizes, thereby weakening subsequent reward prediction errors (Collins and Frank, 2018). Clearly illustrating the need to consider effects of both reinforcement learning and working memory, one study found that apparent learning deficits observed in a group of individuals with schizophrenia were entirely accounted for by working memory’s contribution during instrumental learning on the RLWM task (Collins et al., 2014). Furthermore, once working memory contributions were accounted for, there were no observed learning deficits in the schizophrenia group (Collins et al., 2017). Thus, failing to consider the contributions of working memory may confound the results of studies focusing solely on reinforcement learning.

Recently, Cheng et al. (2024) employed the RLWM framework to disentangle the contributions of working memory and reinforcement learning in mood disorders. The sample consisted of adolescents with either a lifetime history of major depressive (n = 127) or bipolar (n = 28) disorders, or no prior history of psychopathology (n = 62). The results indicated that participants reporting higher severity of current anhedonia exhibited lower reward learning rates. Additionally, those reporting higher severity of current manic symptoms demonstrated reduced contribution of working memory during learning and less stable representations in working memory (Cheng et al., 2024). However, it remains unclear whether similar differences in reinforcement learning and working memory can be observed in adolescent samples that are not specifically enriched with participants with mental disorder diagnoses, but rather characterized by varying levels of symptom burden and, consequently, potential risk of future clinical conditions.

While symptoms of anxiety and depression often increase during adolescence, it is also a time when the brain undergoes major changes in processes that rely on reinforcement learning and working memory (Braams et al., 2015, Casey et al., 2008, Larsen and Luna, 2018, Schreuders et al., 2018, Walker et al., 2017). Thereby, differences in learning across adolescent samples may also stem from differences in developmental maturation, such as age or pubertal maturation. Indeed, both working memory and reinforcement learning exhibit age-related changes throughout adolescence and are believed to follow distinct developmental trajectories. Working memory shows non-linear development, with rapid improvement from late childhood to mid-adolescence (10–15 years old), before stabilizing to adult levels during late adolescence, around 18–20 years old (Ferguson et al., 2021, Tervo-Clemmens et al., 2023). Sensitivity to rewards in the nucleus accumbens, a key reward region in the brain, has shown to follow a quadratic developmental pattern, with a peak during mid-adolescence (Braams et al., 2015, Schreuders et al., 2018, Walker et al., 2017). Also, adolescents may show different learning patterns compared to adults, with prioritization of positive feedback and reduced punishment avoidance learning (Palminteri et al., 2016), as well as overall lower reinforcement sensitivity (Waltmann et al., 2023). However, research on learning rates during adolescence have shown inconsistent findings (Nussenbaum and Hartley, 2019, Wilbrecht and Davidow, 2024), likely partially due to differences in task context across studies (Eckstein et al., 2022).

Investigating the development of reinforcement learning and working memory in instrumental learning, Master et al. (2020) tested participants aged 8–17 (n = 187) and 25–30 (n = 54) on the RLWM task. As neuroimaging-based accounts of brain development during adolescence emphasize the late maturation of the prefrontal cortex and dependent executive functions, including working memory, (Casey et al., 2008, Huizinga et al., 2006, Larsen and Luna, 2018, Steinberg, 2005), Master et al. (2020) hypothesized that while reinforcement learning may be developing, they should observe more protracted development of working memory systems and/or stronger effects of age on working memory compared to reinforcement learning. In addition, they hypothesized that pubertal onset would impact working memory processes, as it has been shown that gonadal hormones affect inhibitory neurotransmission in the prefrontal cortex of rodents (Delevich et al., 2018, Delevich et al., 2019, Juraska and Willing, 2017, Piekarski et al., 2017). The analysis revealed only modest age-related differences in working memory parameters, whereas more protracted changes were observed in reinforcement learning parameters, with a steady increase in learning rate throughout adolescence. These differences were apparent when comparing the age group 8–12 year olds to the older age groups, with no significant differences when comparing 13–17 year olds to the adults. On the other hand, differences in working memory were evident when grouping participants by pubertal stage or hormone levels. Further, in line with the largest age-related differences being evident when comparing pre-adolescents to older participants, Cheng et al. (2024) reported no age-related differences in measures of RL and WM in an older sample of adolescents aged 13–25, with a mean age of 19.8. These findings (Cheng et al., 2024, Master et al., 2020) were somewhat unexpected given previous studies on maturation of working memory and prefrontal regions during adolescence. One possibility is that the original RLWM model lacks the sensitivity to detect subtle developmental differences in working memory.

Recently, the RLWM model used by Master et al. (2020) and Cheng et al. (2024) has been extended and improved to also account for a range of reaction time (RT) effects (McDougle and Collins, 2021). Choice and RT are tightly intertwined during decision making, and by incorporating both, the RLWM-Linear Ballistic Accumulator (RLWM-LBA) model provides a more nuanced account of instrumental learning (McDougle and Collins, 2021). Importantly, by modeling both choice and RT data, identifiability for reinforcement learning and working memory parameters was improved (McDougle and Collins, 2021), in line with previous findings (Ballard and McClure, 2019). The RLWM-LBA has to date not been tested in relation to symptoms of anxiety, depression or age-related differences.

The aims of the current study were threefold. First, building on previous findings suggesting more protracted development in reinforcement learning compared to working memory (Master et al., 2020), we assessed age-related differences in reinforcement learning and working memory among 193 participants aged 12–24 years using a RLWM task and an extended RLWM model, the RLWM-LBA model. The age span in the present study corresponds more closely to the patterns of biological growth and social role transitions that define adolescence in current times, than the traditionally used definition of 10 – 19 years (Sawyer et al., 2018). As previous studies with the RLWM task and original RLWM model showed no age-related differences in reinforcement learning or working memory parameters in participants over 12 years old (Cheng et al., 2024, Master et al., 2020), analyses on age-related differences in reinforcement learning and working memory parameters were exploratory, in which we were interested in whether the extended computational model could identify novel associations. Based on previous work on perceptual decision making showing slower RTs in children and young adolescents compared to adults, along with differences in all parameters from the drift diffusion model, an evidence-accumulation model similar to the LBA (Ratcliff et al., 2012), we expected age-related differences in parameters from the LBA module of the RLWM-LBA model. Specifically, we hypothesized that younger participants would show lower task accuracy and slower RTs, which could be driven by slower rates of information processing (lower drift rates), more noisy decision making (higher start point variability) and/or more cautious response styles (higher response thresholds). As adolescent development may vary between individuals depending on pubertal onset and maturation, we also conducted analyses replacing age with pubertal stage. However, we found that most participants scored at maximum or close to maximum on the measure of pubertal development with little variance, indicating finished puberty. Conclusions drawn from these analyses are therefore unreliable. The results are reported in Supplementary materials.

Second, we tested for associations between the computational parameters and anxiety and depressive symptom burden in our convenience-based sample. Based on previous research using the RLWM task and computational model to identify case-control differences in the use and function of reinforcement learning and working memory in participants with mood disorders (Cheng et al., 2024), we hypothesized that participants that scored high on symptoms of depression and anxiety would show lower learning rates, along with less stable representations in working memory, as reflected in higher decay.

Lastly, to gain a deeper understanding of the complex relations between age, symptoms of depression and anxiety, reinforcement learning and working memory, we ran exploratory, data-driven analyses assessing the value of computational versus behavioral measures for predicting age and symptom burden using machine learning.

## 2 Methods

### 2.1 Participants

**Fig. 1:**
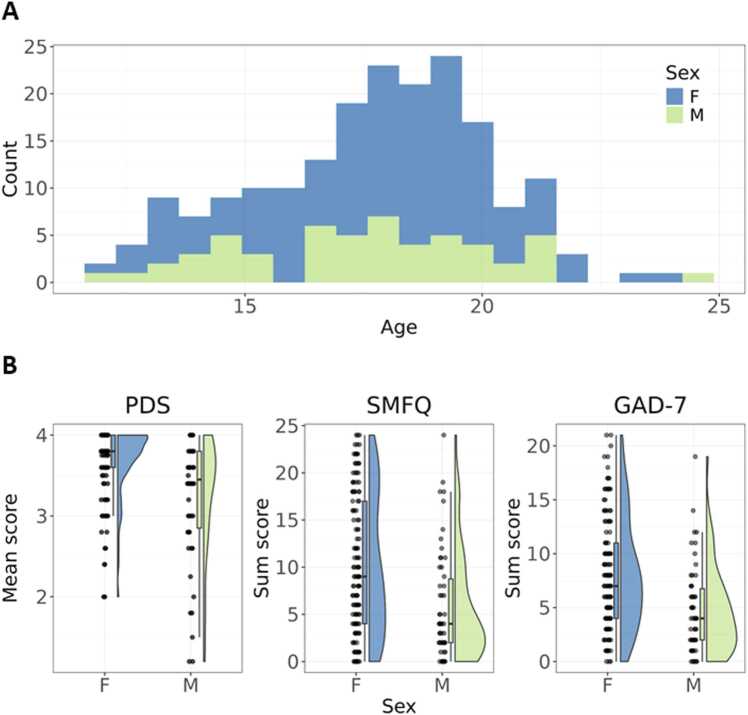
Sample distributions of age, sex, and questionnaire responses. **A**. Age and sex distribution. **B.** Mean score on the measure of pubertal development (PDS), and summed scores on scales measuring depressive symptomology (SMFQ) and general anxiety (GAD-7). F, females; M, males. For distributions on each item of the SMFQ and GAD-7, see Fig. S1-S2.

Participants were drawn from the ongoing Brains and Minds in Transition (BRAINMINT) study in Oslo, Norway. Participants were recruited through social media advertisements and collaborations with other research projects. All participants completed a magnetic resonance imaging (MRI) session, and a sub-sample were invited back to an EEG session, in which the RLWM task was completed after a ten-minute resting state session. 213 participants completed the EEG session. Of these, 193 had also filled out online questionnaires. Thereby, the sample consisted of 193 participants (72 % females, mean ± sd age: 17.76 (2.42), range: 12.35 – 24.31; Fig. 1**A**). Participants’ sex refers to sex registered at birth, which were self-reported when filling out questionnaires and cross-checked with legal sex derived from their national identification number. Inclusion criteria were Norwegian language skills and fulfillment of MRI safety criteria.

The study was approved by the Regional Committee for Medical and Health Research Ethics, South-East Norway (REK, ref. no. 2019/943). All participants, or legal guardians for children below the age of 16, provided informed consent.

### 2.2 Questionnaires

All questionnaires were administered online with the University of Oslo’s survey service, (nettskjema@usit.uio.no).

Pubertal Developmental Scale (PDS, Petersen et al., 1988) is a brief, self-report measure of pubertal status. Participants assess their pubertal development as indexed by height growth, skin changes, and body hair, as well as menarche and breast development in females and voice changes and facial hair in males. Each item is rated on a 4-point scale from 1 (“not started yet”) to 4 (“has completed development”), except for item assessing menarche in females, which was a binary item (1 = “not started”; 4 = “started”). An average score ranging from 1 to 4 was calculated for each participant and used for analyses.

Short Mood and Feelings Questionnaire (SMFQ, Angold et al., 1995) is a 13-item scale designed for affective and cognitive symptoms of depression in childhood and adolescence, derived from the original 33-item Mood and Feelings Questionnaire (MFQ). Due to an oversight in the protocol development of the study, the item “I felt very restless” was not included in the questionnaire that was delivered to the participants. Each item presents a statement related to the previous two weeks, and responses are given on a 3-point Likert scale (0 = “not true”, 1 = “sometimes”, and 2 = “true”). The scale has been shown to have good validity in both early and late adolescence (Angold et al., 1995; Rhew et al., 2010; Turner et al., 2014). The internal reliability of the SMFQ in the present sample was excellent (Cronbach’s alpha =.94).

Generalized Anxiety Disorder Scale (GAD-7, Spitzer et al., 2006) is a 7-item scale for identifying probable cases of generalized anxiety disorder. The questionnaire asks about symptoms of generalized anxiety in the past two weeks and responses are made on a 4-point Likert scale (0 = “not at all”, 1 = “several days”, 2 = “over half the days”, 3 = “nearly every day”). GAD-7 has been shown to have acceptable specificity and sensitivity for detecting clinically significant anxiety symptoms in adolescents (Mossman et al., 2017), and in a systematic review of selected screening tools, the GAD-7 was shown to have the best performance characteristics for identifying generalized anxiety (Herr et al., 2014). Three participants had a missing response on one item each, in which the sample average response on that item was imputed. The internal reliability of the GAD-7 in the present sample was good (Cronbach’s alpha =.88).

### 2.3 The reinforcement learning working memory (RLWM) task

**Fig. 2:**
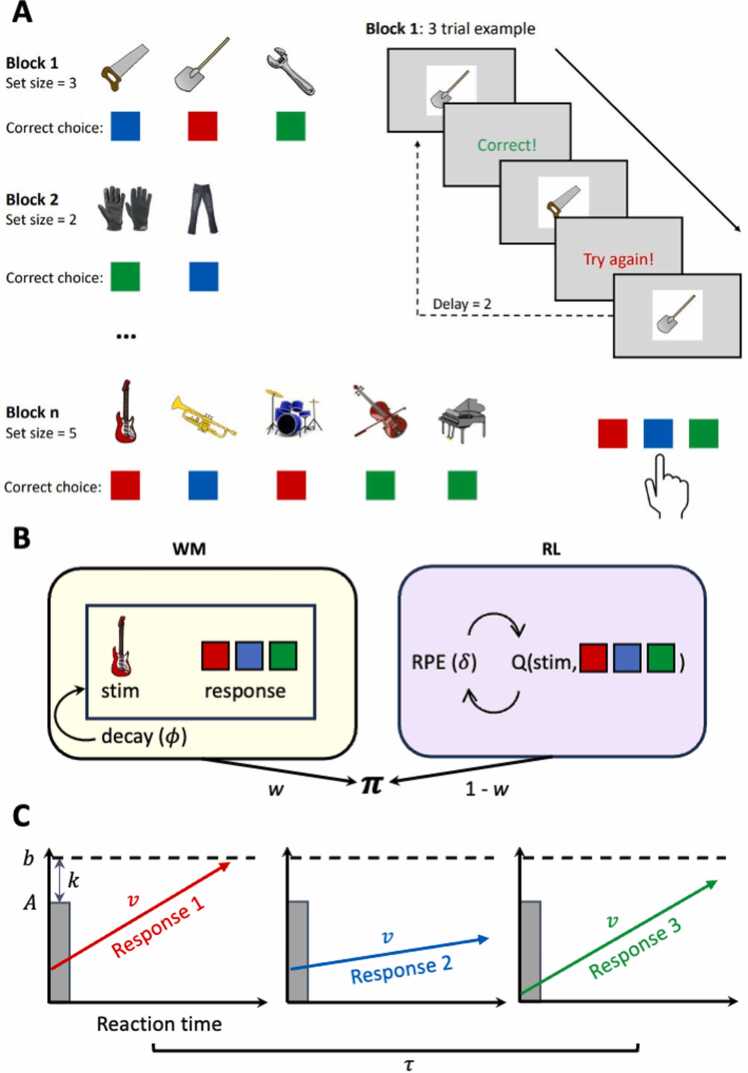
Overview of the RLWM task and RLWM-LBA model. **A.** Participants learn stimulus-action associations by choosing one of three colored buttons followed by immediate, deterministic feedback. **B.** Illustration of the interaction between RL and WM modules in the RLWM model. **C.** Illustration of the LBA model (Brown and Heathcote, 2008).

The RLWM task (Fig. 2**A**) is an instrumental learning task in which participants are instructed to learn the correct stimulus-action associations (Collins and Frank, 2012). We used an adapted version described in Master et al., (2020). Participants completed a training block with two stimuli to familiarize themselves with the task. The training was completed after 15 trials if mean performance in the last ten trials was > 80 % accuracy, otherwise the training continued until the criterion was met, with a maximum amount of 50 trials. This was followed by 468 trials split into 10 blocks, with block unique stimuli. Participants decided when to continue between blocks and the complete task took approximately 15 min depending on the participant’s pace, with no maximum time limit. Before each block, participants were presented with an overview of the upcoming stimuli and encouraged to familiarize themselves with them before starting the block. To tax working memory processes, the number of stimuli to be learned within a block was manipulated, with stimulus set size ranging from two to five stimuli. The set size across blocks was varied, where two had *set size* = 2, three had *set size* = 3, two had *set size* = 4, and three had *set size* = 5. Each visual stimulus was presented 12–14 times in a pseudo-randomized interleaved manner, while controlling for a uniform distribution of delay between two successive presentations of the same stimulus within [1:2*set size] trials, for a total of set size*13 trials. Participants had up to seven seconds to respond by pressing one of three keys. Participants were informed that the same key could be correct for multiple stimuli. Binary, deterministic feedback was presented for 0.75 s immediately after responses, in which correct and incorrect responses were followed by Norwegian translations of “Correct” and “Try again!”, in green and red font, respectively. The feedback was followed by a fixation period of 0.5 s before the next trial. Participants were instructed to respond as quickly and accurately as possible. Failure to respond within seven seconds was indicated by a “No valid answer” message, translated to Norwegian, for 0.75 s. There was no limit on the number of incorrect responses or missed responses that were allowed, and the number of trials was independent of task performance. The block-unique stimuli were from a single category of familiar images, such as clothes, fruits, or instruments, and the category was not shown again in other blocks.

(a) Adapted from Master et al. (2020). (b) Adapted from McDougle and Collins (2021).

The RLWM task was presented using the Psychophysics Toolbox (version 3.0.11; Brainard, 1997) with MATLAB R2014a (The MathWorks, Inc., Massachusetts, USA). Responses were made on a Cedrus RB-740 pad (Cedrus Corporation, San Pedro, CA, USA).

### 2.4 Computational model

We used computational modeling to quantify the processes underlying learning in the RLWM task, and further separate reinforcement learning and working memory. The RLWM model was developed to disentangle the contributions of working memory and reinforcement learning-like processes in learning tasks that previously were modeled only by a reinforcement learning model (Collins and Frank, 2012). The RLWM model consists of reinforcement learning and working memory modules, and model comparison has consistently identified the RLWM model as fitting the observed choice data on the RLWM task better than simpler reinforcement learning models or working memory only models (Collins et al., 2017, Collins et al., 2014, Collins and Frank, 2012, Collins and Frank, 2018), also in adolescent samples (Cheng et al., 2024, Master et al., 2020).

While the RLWM model can separate the contributions of these learning systems to capture processes underlying choices, the model does not account for RT effects. Previous research has identified computational models that combine reinforcement learning models with evidence accumulation models, accounting for both choice and RT data (Fontanesi et al., 2019, Frank et al., 2015, Pedersen et al., 2017, Shahar et al., 2019). In evidence accumulation models (Forstmann et al., 2016), RT and choice is assumed to result from a process where evidence is sampled until a decision boundary for a response option is reached. However, there are a range of choice RT effects that are not addressed by the models that combine RL and evidence accumulation models. These effects show that RT may vary depending on set size (Hick, 1952, Rabbitt, 1968), delay (Hyman, 1953, Remington, 1969), repetition effects (Bertelson, 1965), and interactions between set size and learning (Davis et al., 1961, Mowbray and Rhoades, 1959, Proctor and Schneider, 2018, Schneider and Anderson, 2011). McDougle and Collins (2021) extended the RLWM model with a Linear Ballistic Accumulator (LBA; Brown and Heathcote, 2008) to capture the range of RT effects, while accounting for working memory and instrumental learning processes. In contrast to other commonly used evidence accumulation models, such as the drift-diffusion model (Ratcliff, 1978), the LBA can capture the dynamics of choice in tasks with more than two alternatives.

The aim of the current study was to use the previously developed RLWM-LBA model, referred to as the $\pi_{H}$ model in McDougle and Collins (2021). Model comparison was done with variants of the RLWM-LBA and is reported in the Supplementary materials (Table S1). The RLWM-LBA model, briefly presented below, was identified as the best fitting model.

#### 2.4.1 RL module

The RL module is based on a variant of a standard RL model (Sutton and Barto, 2018), in which learning is driven by the delta rule (Rescorla and Wagner, 1972). According to the delta rule, the expected reward $Q_{\mathrm{rl}}\left(s_{t},a_{t}\right)$ of a stimulus s and action a on trial t is updated based on the prediction error δ from the preceding trial:

(1) $$Q_{t+1}\left(s,a\right)=Q_{t}\left(s,a\right)+\alpha \delta_{t}$$

(2) $$\delta_{t}=r-Q_{t}\left(s,a\right)$$

where α is the learning rate, which quantifies the extent to which new information updates existing value estimates, and r is the reward received.

To account for potential neglect of negative feedback, a bias parameter is included in the RL module. Following negative prediction errors, and thus only on incorrect trials, the learning rate is reduced in proportion to the bias parameter:

(3) $$\alpha =\{\begin{array}{ll} (1-bias)\alpha & if\;\delta_{t}\;<\;0\\ \alpha & if\;\delta_{t}\;\geq \;0 \end{array}$$

The bias parameter is bound between 0 and 1. A value approaching 1 suggests a tendency to neglect negative feedback. Conversely, a bias value of 0 indicates that the participant learns equally from positive and negative prediction errors. Similar studies using RL calculated separate positive and negative learning rates (Frank et al., 2007, Hauser et al., 2015, Lefebvre et al., 2017). However, Master et al. (2020) points out that in previous work with the RLWM model, they consistently found a bias towards learning from positive feedback and parameterizing it as a bias was more efficient than implementing separate learning rates. Also, this approach allows the *bias* parameter to be shared across both RL and WM modules of the RLWM model, which enhances model identifiability (Master et al., 2020).

The softmax choice rule was implemented to capture probability of choosing an action a for a given stimulus s, in which values are transformed into probabilities according to a measure of choice randomness, the inverse temperature β, which was fixed to 50 following McDougle and Collins (2021):

(4) $$P_{\mathrm{RL}}\left(a|,s\right)=\frac{\exp\left(\beta Q_{\mathrm{RL}}\left(s,a\right)\right)}{\Sigma_{i}\exp\left(\beta Q_{\mathrm{RL}}\left(s,a_{i}\right)\right)}$$

where the sum of the denominator is calculated across the three possible actions, $a_{i}$.

#### 2.4.2 WM module

The WM module is based on an understanding of working memory as a system with perfect updating after observed choice outcomes. Like the RL module, the WM module follows the delta learning rule, but with a learning rate of 1:

(5) $$Q_{WM_{t+1}}\left(s,a\right)=Q_{WM_{t}}\left(s,a\right)+\alpha_{\mathrm{WM}}\left(r-{Q_{\mathrm{WM}}}_{t}\left(s,a\right)\right)$$

While the WM module has in principle perfect learning of outcomes in which $Q_{WM_{t+1}}=r_{t}$, neglect of feedback occurs in the WM module as well. Therefore, the bias parameter bias was also calculated for $Q_{\mathrm{WM}}$ (not shown). Working memory is sensitive to short-term forgetting after updating is performed, and therefore sensitive to delays between respective stimuli. Trial-by-trial forgetting of $Q_{\mathrm{WM}}$ is captured by a decay parameter ϕ:

(6) $$Q_{WM_{t}}\left(s_{j},a_{i}\right)=Q_{WM_{t}}(s_{j},a_{i})+\phi \left(\frac{1}{n_{\mathrm{actions}}}-Q_{WM_{t}}\left(s_{j},a_{i}\right)\right)$$

where ϕ pulls $Q_{\mathrm{WM}}$ over all stimuli j and actions i towards their initial value at the start of a trial block, $\frac{1}{n_{\mathrm{actions}}}$, before having observed any outcomes. The task has three choice alternatives, which makes the initial $Q_{\mathrm{WM}}$ value 0.333.

The RL and WM modules have separate action policies, and like the RL module, the probability of choosing an action a for stimulus s is captured by the softmax choice rule, with the same inverse temperature β fixed to 50:

(7) $$P_{\mathrm{WM}}\left(a|,s\right)=\frac{\exp\left(\beta Q_{\mathrm{WM}}\left(s,a\right)\right)}{\Sigma_{i}\exp\left(\beta Q_{\mathrm{WM}}\left(s,a_{i}\right)\right)}$$

The weighting of which module informs choices is described by the mixing value W:

(8) $$P\left(a|,s\right)=WP_{\mathrm{WM}}\left(a|,s\right)+\left(1-W\right)P_{\mathrm{RL}}\left(a|,s\right)$$

where W captures how much working memory contributes to a given choice. Importantly, this depends on an agent’s propensity to use working memory ρ, also referred to as working memory prior weight, and working memory capacity C:

(9) $$W=\rho *\min\left(1,\frac{C}{ns_{k}}\right)$$

Where ns is the set size in a given task block k. C is a value between 2 and 5, and ρ is a proportion between 0 and 1. The influence of working memory on choices is reduced if the set size is beyond working memory capacity C.

#### 2.4.3 LBA module

The standard RLWM model uses softmax transformation to capture choice probabilities based on internal value estimates (Collins and Frank, 2012). In the RLWM-LBA model, choices and associated RT’s are captured with the LBA model. The LBA model conceptualizes choice dynamics between N alternatives as a competitive race in which the winning alternative is the first to cross the decision threshold b. The threshold is the sum of two components, the starting point upper limit A and relative threshold k. The speed of evidence accumulation for each choice is captured by the model variable, drift rate V. The decision process’ starting point is drawn randomly from a uniform distribution within the range of 0 and upper limit A. Additionally, the model accounts for the time required for visually processing a given stimulus and executing a motor response through a non-decision time parameter τ. Following McDougle and Collins (2021), we fixed the non-decision time τ to 0.15, as this was found to improve parameter recovery.

McDougle and Collins (2021) proposed that decision time is influenced not only by the relative difference in stimulus-response associations ($P\left(a|,s\right))$ but also in the uncertainty across all relevant actions, i.e. in how stimulus-response associations vary across stimuli within a block. Motivated by the observation that choice latencies continue to decrease even after choice accuracy reaches asymptotic levels, they incorporated a Shannon entropy transformation (Shannon, 1948) by first averaging the probability of choosing action *i* over all stimuli *k* across all possible states *nS*:

(10) $${\vec{\pi }}_{i}^{\mu }=\frac{1}{\mathrm{nS}}\sum_{i}^{\mathrm{nS}}\pi_{i_{,}k\;}$$

resulting in a single vector of average policies ${\vec{\pi }}_{i}^{\mu }$ with an average action weight for each of the three actions, representing the prior probability of choosing action *i* prior to stimulus presentation. If the average weight of the three actions is similar, uncertainty is assumed to be high, which is linked to longer decision times (Hyman, 1953), while if the same action has the highest action weight across all stimuli, uncertainty is assumed to be low, and decision times should be faster. Following McDougle and Collins (2021), we used Shannon entropy to calculate uncertainty over the average policy ${\vec{\pi }}_{i}^{\mu }$ of choosing action *i*:

(11) $$H_{prior}=-\sum_{i=1}^{3}{{\vec{\pi }}_{i}}^{\mu }\log_{2}\left({{\vec{\pi }}_{i}}^{\mu }\right)$$

Which produces a single trial-specific value $H_{\mathrm{prior}}$. The entropy measure $H_{\mathrm{prior}}$ is incorporated into the calculation of trial-by-trial drift rates by dividing the combined value of $P\left(a|,s\right)$ from the RLWM model (Eq. 8) by $H_{\mathrm{prior}}$. Thus, if entropy is high, representing uncertainty, drift rates for all actions are reduced, resulting in longer decision times. Furthermore, the resulting values are scaled according to a scaling parameter η to ensure value estimates are scaled to capture choice latency.

(12) $$V_{a,t}=\eta \left(\frac{P(a|s)}{H_{\mathrm{prior},t}}\right)$$

Lastly, the LBA probability density function is used to estimate the likelihood of trial-by-trial choice and RT. This is done by using trial-by-trial estimates of internal uncertainties for each of the three response alternatives $V_{a,t}$ together with the LBA parameters starting point upper limit A, relative threshold k, variability in drift rate s and non-decision time τ:

(13) $$\mathrm{Choice}+\mathrm{RT}∼\mathrm{LBA}(k,A,V_{a,t},s,\tau )$$

### 2.5 Model analysis

The RLWM-LBA model was applied to behavioral data from the RLWM task with a hierarchical Bayesian model implemented in STAN via the CmdSTAN package in R (http://r-project.org; R Core Team, 2012). The Bayesian framework allows the use of prior knowledge when defining prior distributions. A hierarchical approach allows estimation of individual subject parameters and the group distribution that they are drawn from simultaneously, which has previously been shown to improve parameter recovery for individual parameter estimates (Ratcliff and Childers, 2015), especially with few data points per subject. Maximum a posteriori (MAP) estimation, which combines prior beliefs and log-likelihood, was used to approximate the mode of posterior distributions.

Weakly informative group priors were set to be in the typical range of estimates for each parameter (McDougle and Collins, 2021). Priors for mean estimates are shown in Table 1. Priors for standard deviations were all set to $\mathrm{lognormal}\left(\log\left(0.5\right),0.35\right)$, except for the standard deviation for working memory capacity C, which was set to $\mathrm{lognormal}(\log\left(.5\right),0.55)$. To constrain the parameters α, ρ, ϕ, and bias between 0 and 1, we approximated individual-level parameters using the normal CDF (via Phi_approx) for the group-level prior. To constrain the working memory capacity parameter C to lie between 2 and 5, subject-level parameters were defined using a shifted and rescaled inverse-logit transform:

(14) $$C=2+3*\mathrm{inv}\_\mathrm{logit}(C_{\mathrm{raw}})$$

**Table 1:** Overview of the free parameters in the RLWM-LBA model with a conceptual description and priors for the mean estimates.

**Free parameters**	**Description**	**Mean estimate priors**
α	Learning rate: how much new information updates existing choice values. Only estimated in the RL module.	$\mu_{\alpha }\;∼\mathrm{normal}(-2,1)$
bias	Positive learning bias: a bias toward learning from positive outcomes and neglecting negative outcomes.	$\mu_{\mathrm{bias}}\;∼\mathrm{normal}(1,1)$
ϕ	Working memory decay: stability of choice values in the WM module. Higher values indicate increased forgetting, as reflected in faster trial-by-trial decay.	$\mu_{\phi \;}∼\mathrm{normal}(-1,1)$
ρ	Working memory prior weight: propensity to use working memory for learning over reinforcement learning.	$\mu_{\rho }\;∼\mathrm{normal}(1,1)$
C	Working memory capacity: working memory’s resource limit. If the current task block’s set size is beyond capacity, working memory is less likely to contribute to learning.	$\mu_{C}\;∼\mathrm{normal}(0,1.5)$
η	Scaling drift rate: combined with the uncertainty corrected choice values to calculate drift rates. Higher values indicate more efficient information processing, as reflected in higher resulting drift rates.	$\mu_{\eta }\;∼\mathrm{normal}(\log(5),0.3)$
A	Start point variability: accumulator starting points are drawn randomly from a uniform distribution [0,A]. Higher upper limits A lead to more stochastic start points, which may result in more incorrect choices and slower RTs.	$\mu_{A}\;∼\mathrm{normal}(\log(1.2),0.35)$
k	Relative threshold: determines the distance from A to the response threshold. Higher values may lead to slower RTs and indicate that more evidence is needed to make a response.	$\mu_{k}\;∼\mathrm{normal}(\log(1),0.3)$

Subject-level parameters were estimated as deviations from the mean group parameter estimate, to ensure exploration of the parameter space for low between-subject variability.

We used the LBA probability density function and random generation function created for Stan (Annis et al., 2017). Inferred latent variables and predicted choice and reaction times were directly calculated in the model and were used as posterior predictive checks (Fig. 4**;** Fig. S3).

Further we conducted parameter recovery analyses to test whether the RLWM-LBA model could reliably recover known parameter values from simulated data. For comparison, we tested parameter recovery for the RLWM model (Collins and Frank, 2012). These results are reported in Supplementary materials (Fig. S4).

### 2.6 Statistical analyses

Statistical analyses were done in R version 4.2.0 (http://r-project.org; R Core Team, 2012). For all statistical analyses linking behavioral and computational model-dependent variables to other variables of interest, a Bayesian regression approach was taken using the brms package (Bürkner, 2017, 2018) in R. Bayes factors (BFs) were calculated using the Savage-Dickey density ratio method (Wagenmakers et al., 2010). The BF reflects the ratio of marginal likelihoods between two competing hypotheses, the alternative and null hypothesis, and can be interpreted as the strength of evidence in favor of either. The reported BFs were calculated against a null hypothesis that assumes the resulting coefficient will equal 0, with BFs below 1 indicating evidence in favor of the alternative hypothesis. The following BF_01_-values can be interpreted as evidence for the alternative hypothesis: 0.3–1 (anecdotal), 0.1–0.3 (moderate), 0.03–0.1 (strong), 0.01–0.03 (very strong), < 0.01 (extreme). BF_01_> 1 indicates evidence towards the null hypothesis: 1–3 (anecdotal), 3–10 (moderate), 10–30 (strong), 30–100 (very strong), > 100 (extreme) (Jeffreys, 1961, Wagenmakers et al., 2018). For all Bayesian regression models, weakly regularizing priors normally distributed around zero (mean = 0, SD = 1) were used for all coefficients to reduce the probability of false positives and improve model convergence. Also, for each Bayesian regression model, four Markov chain Monte Carlo samplers of 10,000 iterations each, including 5000 warm-up samples, were used. The R-hat values were smaller than 1.02 for all estimations, indicating satisfactory convergence of the chains. All independent variables were treated as continuous and standardized before analyses, except for sex which was treated as a factor with 0 = Female and 1 = Male.

#### 2.6.1 Behavioral level analyses

**Fig. 3:**
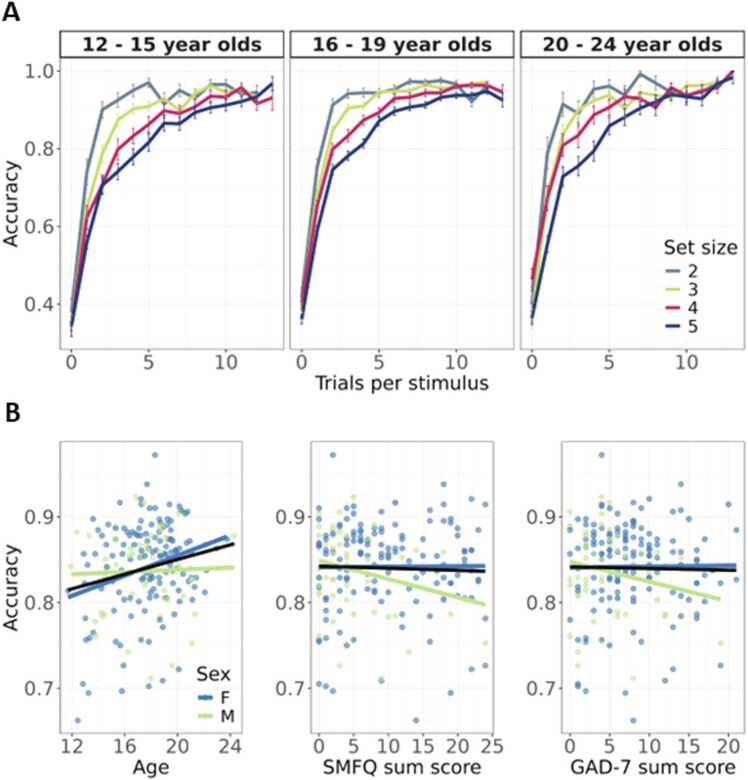
Task performance on the RLWM task **A.** Observed learning curves at different set sizes, split into approximately evenly spaced age groups. Each stimulus was presented 12–14 times within a block. **B.** Overall task performance and relation to age, SMFQ and GAD-7 sum scores, by sex. For visualization purposes lines show the predicted accuracy from a linear model. The black line shows mean performance for all participants. F, females; M, males.

Learning trajectories were visualized by collapsing participants’ accuracy as a function of the number of encounters with given stimulus, or stimulus iterations, in which each stimulus was repeated 12–14 times within a block (Fig. 3**A**). Also, we visualized high-level relations between task performance and age, SMFQ sum score, GAD-7 sum score, and sex. We plotted mean accuracy across all trials as a function of age and symptoms scores separately, while accounting for sex-differences, using simple linear regression models (Fig. 3**B**).

To assess differences in task performance relating to age, symptoms of depression and generalized anxiety, we fit a mixed effects logistic regression model with a Bernoulli likelihood and logit link predicting accuracy, 0 or 1, at trial-level data with subject-level random intercepts. We modeled accuracy as a function of main effects and interactions involving age, GAD-7 sum score, SMFQ sum score, and sex, as well as task factors. The task factors were delay (number of trials since last presentation of the same stimulus), set size, and reward history (cumulative number of rewards for the same stimulus). Task block was also included to control for overall task improvement and to see if there were signs of fatigue. All independent variables were standardized. We report the odds ratio for interpretability, exponentiated from the estimates on a log odds scale, as well as exponentiated 95 % credible intervals (CI) and BF_01_’s. The 95 % CI represents the range within which the true value of the parameter lies with 95 % probability, given the data and the model. Full model output can be found in Supplementary materials (Table S2).

#### 2.6.2 Computational level analyses

To assess linear associations between age and model parameters, Bayesian regression models were fit with age as dependent variable, all model parameters as independent variables, and sex as interaction term to control for potential diverging associations between males and females.

Next, we assessed links between RLWM-LBA parameters and scale responses, using Bayesian ordinal regression models on the single item level, meaning that we modeled each item response from the PDS, GAD-7, and SMFQ scales separately as ordinal outcomes rather than using sum scores. While metric methods such as linear regression are frequently used for analyzing ordinal data, such approaches may produce false positives, failure to detect effects, or distorted effect size estimates (Bürkner and Vuorre, 2019, Liddell and Kruschke, 2018). Metric models assume that the dependent variable is continuous and that there are equal intervals between the discrete response levels, an assumption that is not met when dealing with ordinal data such as the PDS, GAD-7 and SMFQ responses. Therefore, we used the cumulative logit model, with scale item response as dependent variable and random intercepts for each subject.

To test for maturation-related differences in associations with the RLWM-LBA-parameters that were picked up by pubertal maturation rather than age, we used Bayesian ordinal regression with PDS item response as the dependent variable and RLWM-LBA parameters as independent variables. Because the female and male participants respond to different items of the PDS, we used regression models on females and males separately. These results are reported in Supplementary materials (Fig. S12**,** Table S3**-4**).

To assess the link between RLWM-LBA parameters and scale responses measuring symptoms of generalized anxiety and depression, we tested Bayesian ordinal regression models with item response on the GAD-7 or SMFQ as dependent variable. The independent variables were the RLWM-LBA parameters, controlling for interaction effects with sex, and including covariates age and PDS mean score to control for potential effects arising from differences in age and pubertal maturation.

Posterior predictive checks from Bayesian regression models are shown in Supplementary materials (Fig. S9**-11**), as well as full model output (Table s5**-7**).

Lastly, to compare the relevance and sensitivity to relevant individual variables, we examined the value of the RLWM-LBA parameters versus RLWM task behavioral measures for predicting age and SMFQ and GAD-7 sum scores. This was implemented in Python version 3.9.6 with scikit-learn (Pedregosa et al., 2011). Employing an approach from similar studies (Pedersen et al., 2021, Tichelaar et al., 2025, Wiecki et al., 2015), the data was split into training and test sets with an 80 %/20 % split stratified on the outcome variable, followed by a 10-fold cross-validation to find the best hyperparameters, before testing the trained model on the held-out test set. This process of splitting, cross-validation, training, and testing was repeated 100 times. We trained ridge regression models, which regularize coefficients towards zero, and can handle potential collinearity among predictors. The performance metrics reported were calculated as means across 100 iterations of model training and testing. Behavioral variables included accuracy at each set size, overall accuracy, and mean reaction time across set sizes on successful trials.

## 3 Results

### 3.1 Behavioral results

Participants across all age groups learned the correct stimulus-action associations (Fig. 3**A**). The Bayesian mixed effects logistic regression model showed extreme evidence in favor of the task factors affecting trial-level performance. Increasing reward history was associated with higher odds of correct response (OR = 5.49, 95 % CI = [5.49–5.96], BF_01_ = <.00), whereas increasing set size and delay were associated with lower odds of correct response (Set size: OR =.78, 95 % CI = [.76–.8], BF_01_ = <.00; Delay: OR =.94, 95 % CI = [.91–.96], BF_01_ = <.00). There were also signs of improvements as the number of completed task blocks increased (OR = 1.09, 95 % CI = [1.06–1.12], BF_01_ = <.00).

Further, the model showed moderate evidence in favor of a positive association between age and trial-level performance (OR = 1.13, 95 % CI = [1.05–1.23], BF_01_ =.26) (Fig. 3**B**). There was anecdotal evidence for no sex differences in performance (OR =.87, 95 % CI = [.73–1.02], BF_01_ = 2.8). Although the model indicated no interaction between age and sex (OR =.9, 95 % CI = [.78–1.04], BF_01_ = 4.9), the age-related improvement in performance seems to be driven by the female group (Fig. 3**B**). There was strong evidence for no interaction effect between age and symptom sum scores (Age x SMFQ: OR = 0.99, 95 % CI = [.89–1.1], BF_01_ = 18; Age x GAD-7: OR =.96, 95 % CI = [.87–1.07], BF_01_ = 14.35), as well as strong to very strong evidence for no interactions between age and task factors, with BF_01_’s ranging from 21.61 (Age x Reward history) to 69.46 (Age x Task block).

There was strong evidence for no association between symptom sum scores and trial-level performance (SMFQ: OR =.98, 95 % CI = [.88–1.1], BF_01_ = 15.67; GAD-7: OR = 1, 95 % CI = [.89–1.13], BF_01_ = 16.38). There was anecdotal evidence for an interaction between SMFQ sum score and task block, indicating that the task block-related improvement in performance was smaller for participants that scored higher on the SMFQ (OR =.94, 95 % CI = [.91–.98], BF_01_ =.57). There was moderate evidence towards no interaction between SMFQ sum and sex (OR =.89, 95 % CI = [.7–1.13], BF_01_ = 4.94), as well as between GAD-7 sum and sex (OR = 1.03, 95 % CI = [.81–1.30], BF_01_ = 7.9). There was strong to very strong evidence for no interaction between SMFQ sum and the other task factors, with BF_01_’s ranging from 16.02 (SMFQ sum x Reward history) to 39.23 (SMFQ sum x Delay). Lastly, there was moderate to very strong evidence for no interaction between GAD-7 sum and neither of the task factors, ranging from 8.15 (GAD-7 sum x Task block) to 48.48 (GAD-7 sum x Delay). The full model output is summarized in Table S2.

### 3.2 Model fit

**Fig. 4:**
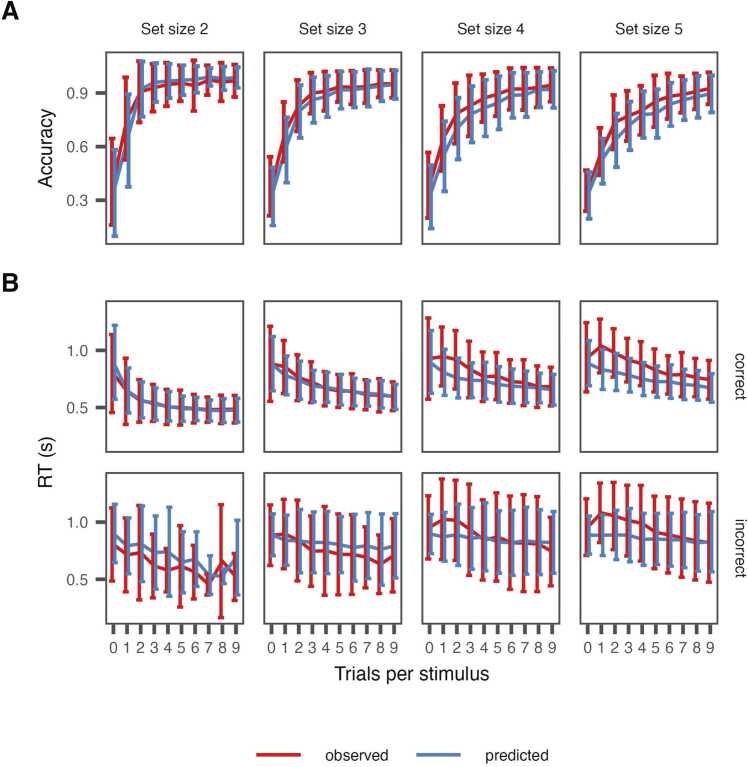
Model fit. Observed and RLWM-LBA model predicted results for A) accuracy and B) RT for correct and incorrect choices. RT, reaction time.

Trial-by-trial choice and RT data was analyzed with a hierarchical Bayesian version of the RLWM-LBA model (McDougle and Collins, 2021). Model predicted data showed good fit with the observed data (Fig. 4), capturing the observed increased accuracy and decreased response time with stimulus iterations, and how these effects varied across set size.

### 3.3 Association between RLWM-LBA model parameters and age

**Fig. 5:**
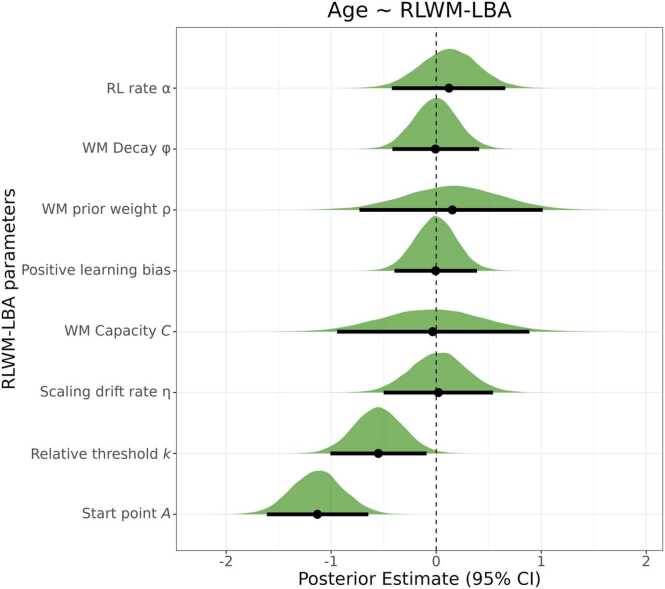
Posterior distributions for the associations between RLWM-LBA parameters and age. Points represent the mean estimate for each association and error bars represent the 95 % CI.

Fig. 5 shows posterior distributions reflecting associations between RLWM-LBA parameters and age. The starting point distribution parameter, A, showed a very strong association with age (β = −1.13, 95 % CI = [-1.612 to −.65], BF_01_ = <.00), indicating a reduction in starting point variability with increasing age. There was moderate evidence for a negative association between the relative threshold k and age (β = −.55, 95 % CI = [-1 to −.09], BF_01_ =.26). Older participants showed lower relative threshold values, and thus shorter distances from the upper limit of the start point distribution to the response threshold.

The remaining BF_01_’s showed evidence for no associations between RLWM-LBA parameters and age, ranging from 2.1 (Working memory prior weight ρ) to 5.22 (Positive learning bias), and no interactions between RLWM-LBA parameters and sex, ranging from 1.085 (Sex x Start point A) to 2.66 (Sex x Positive learning bias). The results are summarized in Table S5 and all posterior distributions are shown in Fig. S13.

### 3.4 Association between RLWM-LBA model parameters and anxiety and depression scales

**Fig. 6:**
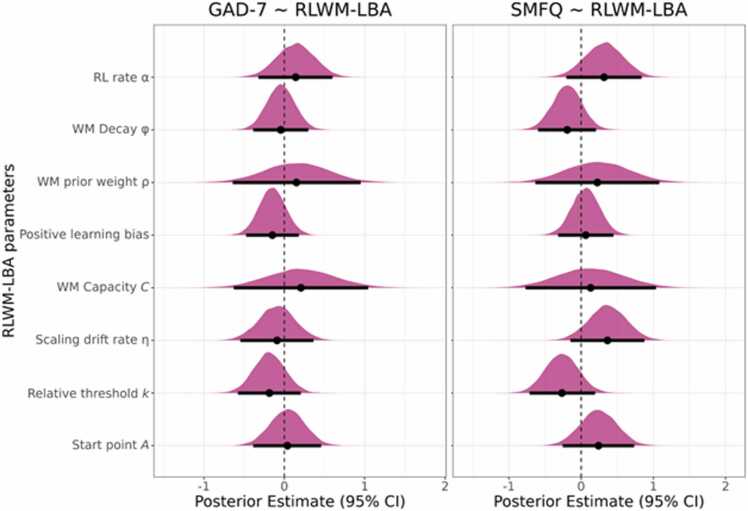
Posterior distributions for the main effects of RLWM-LBA parameters on GAD-7 and SMFQ item responses. Points represent the mean estimate for each association and error bars represent the 95 % CI.

The posterior distributions for the associations between RLWM-LBA parameters and scale responses are shown in Fig. 6. Briefly, there were no strong associations between RLWM-LBA parameters and GAD-7 responses, with BF_01_’s ranging from 2.1 for working memory capacity to 5.5 for working memory decay (Table S6), indicating anecdotal to moderate evidence for no association. Similarly, BF_01_’s showed evidence for no association between RLWM-LBA parameters and SMFQ responses (Table S7), ranging from anecdotal (scaling drift rate: BF_01_ = 1.49) to moderate evidence (positive learning bias: BF_01_ = 4.82).

### 3.5 Predictive value of RLWM-LBA parameters

Next, we assessed the value of individual parameter estimates from the RLWM-LBA model and observed behavioral measures for predicting age using regularized linear regression and 10-fold cross-validation, testing the model on held-out data. The metrics from 100 models were averaged.

**Fig. 7:**
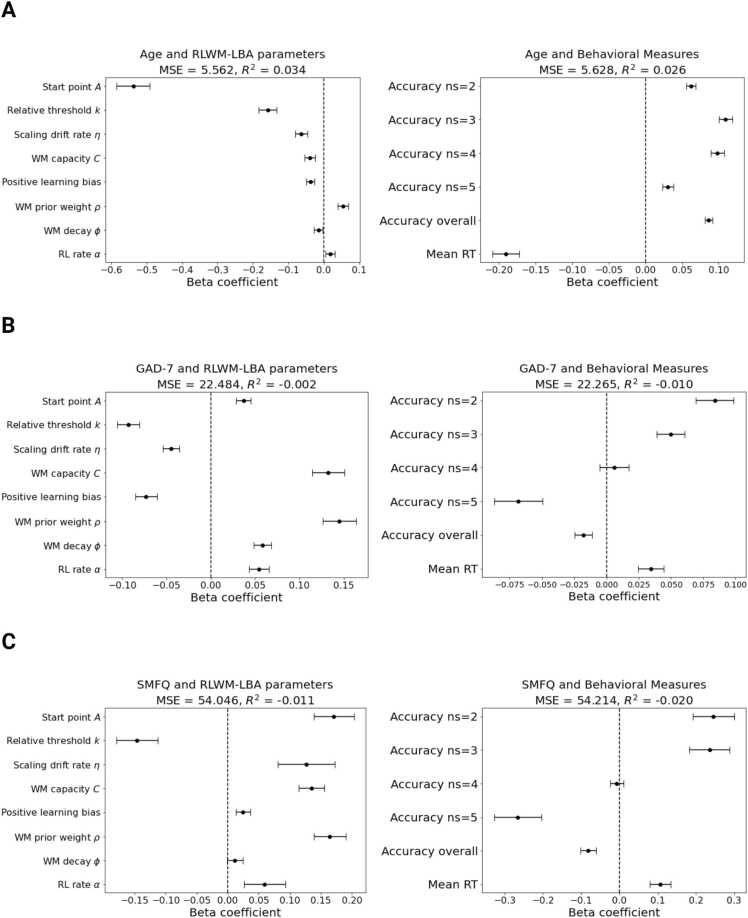
Predictive value of RLWM-LBA parameters versus observed behavioral measures of accuracy and reaction time for age and symptom burden of depression and generalized anxiety. **A.** Mean estimated beta-coefficients from the linear age prediction model with 95 % confidence intervals. Predictors are shown on the y-axis. **B-C.** Mean estimated beta-coefficients from the linear GAD-7 and SMFQ sum score prediction models with 95 % confidence intervals. MSE, Mean squared error.

The RLWM-LBA parameters showed poor predictive value, with a mean squared error (MSE) of 5.562 (SD =.59, range = [3.96–7.36]) and r^2^ of.034 (SD =.08, range = [-.26–.16]). The starting point parameter, A, showed the largest absolute distance from 0, implicating it as the feature with highest importance for age prediction (Fig. 7**A**). Observed accuracy and reaction time on the RLWM task similarly poor predictive value for age (MSE = 5.63, SD =.42, range = [4.26–6.5]; r^2^ =.026, SD =.045, range = [-.11–.12]), with mean reaction time on correct trials showing largest weight (Fig. 7**A**).

Neither RLWM-LBA parameters nor behavioral variables performed well as predictors of symptom burden (Fig. 7**B-C**). The RLWM-LBA parameters predicting GAD-7 sum score showed a MSE of 22.48 (SD = 1.49, range = [18.68–28.11]) and r^2^ of −.002 (SD =.03, range = [-.18–.04]), whereas behavioral measures performed similarly (MSE = 22.27, SD = 1.5, range = [19.29–25.67]; r^2^ = −.01, SD =.02, range = [-.14–.007]). When predicting SMFQ sum scores, performance was similarly poor for RLWM-LBA parameters (MSE = 54.05, SD = 2.96, range = [47.03–67.36]; r^2^ = −.011, SD =.04, range = [-.28–.015]) and behavioral measures (MSE = 49.83, SD = 2.82, range = [47.5–63.17]; r^2^ =.02, SD =.04, range = [-.19–.02]).

## 4 Discussion

The current study aimed to assess age-related differences in working memory and reinforcement learning processes in adolescents aged 12–24, as well as differences in relation to symptoms of generalized anxiety and depression. Using the RLWM task, we found that older adolescents performed better. Further, we found signatures of reinforcement learning and working memory processes on the behavioral level, with the task factors delay and set size showing negative effects and reward history showing positive effects on task performance. Also, participants displayed learning trajectories resembling those seen in previous research with the RLWM task (Fig. 3**A**; Collins and Frank, 2012; Master et al., 2020), in which learning was faster in lower set sizes likely relying on working memory. To gain insight into possible mechanisms underlying differences in performance, we applied the computational RLWM-LBA model. In line with our hypothesis, we found age-related differences in LBA parameters, specifically a strong age-related decrease in start point parameter A, indicating less variable response initiation among the older adolescents. Also, we found moderate evidence for an age-related decrease in relative thresholds k, indicating speeded decision making with age (Fig. 5). We expected higher symptom burden of depression or generalized anxiety to be associated with lower learning rates and larger decay of information in working memory. However, we found no differences in task performance depending on symptoms of depression or generalized anxiety, as well as no links to RLWM-LBA parameters (Fig. 6). Overall, most associations between model parameters and age (Fig. 5), as well as model parameters and SMFQ or GAD-7 responses (Fig. 6), were small. Lastly, we assessed the predictive value of the RLWM-LBA model parameters versus behavioral measures of task performance, yielding low performance for predicting age or symptom burden of depression or generalized anxiety.

### 4.1 Age-related differences in RLWM-LBA

In the Bayesian regression analysis, we found strong evidence for a negative association between age and the start point variability parameter, A (Fig. 5). In the LBA, accumulators’ start points are randomly drawn from a uniform distribution of the form [0, A] (Fig. 2**C**). Such an age-related decrease in start points, indicate that older participants have more narrow distributions in which start points may be drawn from and thereby less variable initial points of evidence accumulation. With high A responses may be more equally divided between correct and incorrect responses, depending on which accumulator sampled the high start point (Brown and Heathcote, 2008). This may contribute to the observed age-related increase in overall accuracy (Fig. 3**B**). The effect may be more pronounced under difficult task conditions, as the start point parameter showed a larger, negative correlation with accuracy in the highest set size (Rho = −.37) compared to the lowest set size (Rho = −.18; Fig. S8). Further, we found evidence for a negative association between relative threshold and age, although this was a moderate association. In the LBA model (Brown and Heathcote, 2008), higher relative thresholds contribute to slower RTs, as responses need more evidence to reach response thresholds, whereas higher start points contribute to both slower RTs and more incorrect responses. We observed an age-related decrease in RT on the RLWM task (Fig. S7), which may be explained by the combination of lower start point variability and response thresholds, reflecting improvements in speeded decision-making.

Most research on age differences in evidence accumulation model parameters have focused on the drift diffusion model (Ratcliff, 1978), whereas research using the LBA is limited (Theisen et al., 2021). Furthermore, the studies that have used the LBA have typically compared young and older adults to understand age-related slowing. For example, Forstmann et al., (2011) compared older (61–79 years) and younger (22–32 years) adults using a perceptual decision-making task in which participants were instructed to respond either quickly or accurately. In addition to differences in other LBA parameters, they found on average higher starting point variability in the older compared to the younger participants, and concluded that the older adults show less proficiency in controlling their bias or response caution settings (Forstmann et al., 2011).

The age-related decrease in start point variability, and thus choice stochasticity, may be indicative of a decrease in explorative tendencies. Indeed, several studies have shown that children, display more stochastic choices indicative of exploration (Gopnik, 2020, Nussenbaum and Hartley, 2019, Sumner et al., 2019), whereas adults often show exploitative tendencies where they make choices based on prior knowledge that are known to lead to rewards. While random exploration can be an effective learning strategy in many environments (Gopnik, 2020, Sutton and Barto, 2018, Wilson et al., 2014), the RLWM task used here does not reward noisy choices (i.e. stochastic choices that are not driven by reward or information) as outcomes are deterministic and binary throughout the task blocks. The task prompts exploration in early stages of a task block in which the correct stimulus-action associations are unknown, and a shift towards an exploitative tendency as the participant learns. Failing to control explorative tendencies and disregarding learned choice values, leads to lower task performance. While higher stochasticity in start points may be related to increased explorative tendencies, we note that the RLWM task and RLWM-LBA model used here are not designed for nuanced accounts of explore-exploit tendencies.

Previous accounts of adolescent development have placed weight on the protracted development of the prefrontal cortex (Casey et al., 2008, Larsen and Luna, 2018) and prefrontal cortex-dependent executive functions, including working memory (Ferguson et al., 2021, Huizinga et al., 2006, Steinberg, 2005, Tervo-Clemmens et al., 2023). While reinforcement learning may still be developing during adolescence, it may be expected that the largest age-related differences would be observed in working memory measures on the RLWM task compared to reinforcement learning measures. As this was not seen in previous work with the RLWM task and model, one of our aims was to assess whether a more comprehensive, and possibly more sensitive, model, the RLWM-LBA, would show different results. Although parameter recovery was improved for all parameters, except for positive learning bias, when comparing the RLWM and RLWM-LBA (Fig. S4), we did not find evidence for associations between age and reinforcement learning or working memory parameters. We note that parameter recovery for our RLWM-LBA was worse than McDougle and Collins (2021) for some parameters, which was likely due to the use of different versions of the RLWM task, in which McDougle and Collins (2021) used an extended version including task blocks with set sizes 1 and 6 and therefore nearly twice as many trials, whereas we used the RWLM task adapted for adolescents with fewer trials and maximum set size 5.

Our observed age-related associations with model parameters deviated from previous research on age-related differences with the RLWM task and model (Master et al., 2020). For example, Master et al. (2020) observed an increase in learning rate, which was most pronounced when comparing the 8 – 12 year olds to the older participants, whereas our Bayesian regression models did not show evidence in favor of such an association. These differences are likely due to different sample characteristics, where our adolescent sample lacks 8 – 11 year olds and has an emphasis on later adolescence, ranging from 12 to 24, with most participants within the ages 17 – 19 (Fig. 1**A**). In line with our findings, Cheng et al. (2024) found no significant age-related differences in RLWM parameters using a similar RLWM model as Master et al. (2020).

When assessing associations between RLWM-LBA parameters and pubertal status, we found strong evidence for a negative association between pubertal maturation and the starting point variability parameter in the female group (Fig. S12**;** Table S3), in line with the association between age and RLWM-LBA parameters. Maturational differences in learning processes during childhood and early adolescence may be better captured by measures of biological maturation, e.g. puberty status, rather than age, especially when comparing females and males. Therefore, we used Bayesian ordinal regression models in which age was replaced by item responses on the PDS separably for males and females. However, these results should be interpreted with caution. Most of the sample score at close to maximum or at maximum level on the PDS, indicating completed pubertal maturation (Fig. 1**B**). There were sex differences in pubertal maturation and there were slight indications of sex differences in associations between puberty and RLWM-LBA parameters (Fig. S12). Yet, the male sample is relatively small, limiting the ability to draw firm conclusions regarding sex differences and the BFs indicated no association between puberty and RLWM-LBA parameters in the male sample.

### 4.2 Associations between RLWM-LBA and symptoms of anxiety and depression

As differences in reinforcement learning and working memory processes have previously been identified using the RLWM task and model in adolescents with histories of mood disorders (Cheng et al., 2024). We tested if similar differences could be detected based on symptom burden in an adolescent sample that was not enriched with participants with mental disorder diagnoses. We hypothesized that higher symptom sum scores would be associated with lower learning rate and less stable representations in working memory, as reflected in higher working memory decay. Responses on the GAD-7 and SMFQ show that some of the adolescents had elevated symptom burden (Fig. 1**B**).

On the behavioral level, the Bayesian regression model indicated evidence for no association between GAD-7 and SMFQ sum scores and task performance. Next, we assessed associations between the RLWM-LBA parameters and item-level responses on the scales using Bayesian ordinal regression models (Fig. 6). In line with the lack of differences in task performance, there were no strong associations between RLWM-LBA parameters and GAD-7 or SMFQ responses, with BF_01_’s indicating evidence for no associations. We note that these were anecdotal to moderate evidence, rather than very strong or extreme evidence in favor of no association, which may be related to the sample size. The difference in results between our study and Cheng et al. (2024) may reflect challenges in transferring case-control findings to samples drawn from the general population, in which the latter often requires a large sample size for a fully powered investigation of potentially subtle associations between cognition and dimensions of psychopathology.

The lack of associations between RLWM-LBA parameters and GAD-7 responses partially converge with a recent study assessing RL and WM processes in relation to physiological and cognitive anxiety levels (Senta et al., 2025). Senta et al. (2025) used an RLWM model and found reduced learning rate and higher rate of WM decay in participants that reported higher physiological anxiety scores. On the other hand, they found no significant associations between RLWM parameters and levels of cognitive anxiety or worrying, or symptoms of depression. While it would be interesting to test for a distinction between cognitive and physiological anxiety scores in our sample, the GAD-7 mixes both cognitive and physiological symptoms of generalized anxiety and a split between the two would result in too few items per category.

### 4.3 Limitations

The current findings should be interpreted considering relevant study limitations. The age distribution of the sample is not even, with relatively few participants in the younger age range, thereby limiting the ability to capture age-related differences. Further, most of the participants were female (72 %). Males and females typically follow different maturational trajectories during adolescence, with females tending to begin puberty earlier than their male peers. While it would be interesting to investigate how sex dependent maturational differences correlate with cognitive differences, the relatively small sample of males restricts the potential to assess such sex-differences. Additionally, cross-sectional study designs are not well-suited for examining the dynamic changes that occur during adolescence and future research should leverage longitudinal designs. Also, only a few males scored highly on symptoms of depression or generalized anxiety, so analyses related to symptoms were even further skewed towards female participants. Thus, any investigation of sex-differences in associations between symptoms and RLWM-LBA parameters is limited. Further, although several participants reported high levels of generalized anxiety and depressive symptoms, we currently do not have information on whether any of these participants have a formal diagnosis of depression or generalized anxiety disorder. Future BRAINMINT studies will be able to leverage Norwegian electronic health registry data, including psychiatric diagnoses, to test for associations with current and future diagnosis, thereby enhancing the relevance for clinical prediction. Lastly, although the values were coherent with widely accepted values for working memory capacity (Cowan, 2010), the working memory capacity parameter showed little variance, likely due to over-regularization towards the group mean (Fig. S4). Also, some parameters showed poor (Fig. S4), specifically working memory decay and positive learning bias. We observed evidence for associations with start point variability and relative threshold, which both showed good recovery (Fig. S4). However, the potential for discovering associations with poorly recovered parameters is limited and evidence of no associations with age and symptom sum scores should be interpreted thereafter.

In conclusion, this study investigated age-related differences in working memory and reinforcement learning in adolescents, along with their relationships to symptoms of depression and generalized anxiety. Overall task accuracy improved with age, which may be explained by less stochastic decision making, as reflected in the negative association between start point variability and age. On the other hand, we did not find evidence for differences in task performance depending on symptoms of depression or generalized anxiety, accompanied by evidence for no associations between symptoms and RLWM-LBA parameters. Further research is required to replicate these findings in larger, more diverse cohorts and to explore the relation between RLWM-LBA parameters and symptoms of psychopathology.

## CRediT authorship contribution statement

**Pedersen Mads Lund:** Writing – review & editing, Visualization, Supervision, Methodology, Formal analysis, Data curation, Conceptualization. **Westlye Lars Tjelta:** Writing – review & editing, Supervision, Project administration, Methodology, Funding acquisition, Conceptualization. **Collins Anne G. E.:** Writing – review & editing, Methodology. **Frogner Erik Rimestad:** Writing – original draft, Visualization, Methodology, Investigation, Formal analysis, Data curation, Conceptualization. **Torgeir Moberget:** Writing – review & editing. **Rikka Kjelkenes:** Writing – review & editing, Investigation. **Andreas Dahl:** Writing – review & editing, Investigation.

## Declaration of generative AI and AI-assisted technologies in the writing process

During the preparation of this work the author(s) used ChatGPT (OpenAI) in order to explore alternative wording or phrasing for segments of their own-written sentences. After using this tool/service, the authors reviewed and edited the content as needed and take full responsibility for the content of the publication.

## Declaration of Competing Interest

The authors declare that they have no known competing financial interests or personal relationships that could have appeared to influence the work reported in this paper.

## Data Availability

The code used in the study and a synthetic version of the data is available in a public repository (Open Science Framework) (https://osf.io/vq62u/). Sharing of the raw data requires a data transfer agreement.

## References

[bib1] Abramovitch A., Short T., Schweiger A. (2021). The c factor: cognitive dysfunction as a transdiagnostic dimension in psychopathology. Clin. Psychol. Rev..

[bib2] Angold A., Costello E.J., Messer S.C., Pickles A. (1995). Development of a short questionnaire for use in epidemiological studies of depression in children and adolescents. Int. J. Methods Psychiatr. Res..

[bib3] Annis J., Miller B.J., Palmeri T.J. (2017). Bayesian inference with stan: a tutorial on adding custom distributions. Behav. Res. Methods.

[bib4] Asselmann E., Wittchen H.-U., Lieb R., Beesdo-Baum K. (2018). Sociodemographic, clinical, and functional long-term outcomes in adolescents and young adults with mental disorders. Acta Psychiatr. Scand..

[bib5] Auerbach R.P., Pagliaccio D., Hubbard N.A., Frosch I., Kremens R., Cosby E., Jones R., Siless V., Lo N., Henin A., Hofmann S.G., Gabrieli J.D.E., Yendiki A., Whitfield-Gabrieli S., Pizzagalli D.A. (2022). Reward-Related neural circuitry in depressed and anxious adolescents: a human connectome project. J. Am. Acad. Child Adolesc. Psychiatry.

[bib6] Aylward J., Valton V., Ahn W.-Y., Bond R.L., Dayan P., Roiser J.P., Robinson O.J. (2019). Altered learning under uncertainty in unmedicated mood and anxiety disorders. Nat. Hum. Behav..

[bib7] Balderston N.L., Vytal K.E., O’Connell K., Torrisi S., Letkiewicz A., Ernst M., Grillon C. (2017). Anxiety patients show reduced working memory related dlPFC activation during safety and threat. Depress Anxiety.

[bib8] Ballard I.C., McClure S.M. (2019). Joint modeling of reaction times and choice improves parameter identifiability in reinforcement learning models. J. Neurosci. Methods.

[bib9] Beck A.T. (2008). The evolution of the cognitive model of depression and its neurobiological correlates. Am. J. Psychiatry.

[bib10] Beevers C.G., Worthy D.A., Gorlick M.A., Nix B., Chotibut T., Maddox W.T. (2013). Influence of depression symptoms on history independent reward and punishment processing. Psychiatry Res..

[bib11] Bertelson P. (1965). Serial choice Reaction-time as a function of response versus Signal-and-Response repetition. Nature.

[bib12] Bishop S.J., Gagne C. (2018). Anxiety, depression, and decision making: a computational perspective. Annu. Rev. Neurosci..

[bib13] Bornstein A.M., Daw N.D. (2012). Dissociating hippocampal and striatal contributions to sequential prediction learning. Eur. J. Neurosci..

[bib14] Bornstein A.M., Norman K.A. (2017). Reinstated episodic context guides sampling-based decisions for reward. Nat. Neurosci..

[bib15] Braams B.R., Duijvenvoorde A.C.K. van, Peper J.S., Crone E.A. (2015). Longitudinal changes in adolescent Risk-Taking: a comprehensive study of neural responses to rewards, pubertal development, and Risk-Taking behavior. J. Neurosci..

[bib16] Brainard D.H. (1997). The psychophysics toolbox. Spat. Vis..

[bib17] Brown S.D., Heathcote A. (2008). The simplest complete model of choice response time: linear ballistic accumulation. Cogn. Psychol..

[bib18] Bürkner P.-C., Vuorre M. (2019). Ordinal regression models in psychology: a tutorial. Adv. Methods Pract. Psychol. Sci..

[bib19] Casey B. j, Jones R.M., Hare T.A. (2008). The adolescent brain. Ann. N. Y. Acad. Sci..

[bib20] Chavez-Baldini U., Nieman D.H., Keestra A., Lok A., Mocking R.J.T., de Koning P., Krzhizhanovskaya V.V., Bockting C.L.H., van Rooijen G., Smit D.J.A., Sutterland A.L., Verweij K.J.H., van Wingen G., Wigman J.T.W., Vulink N.C., Denys D. (2023). The relationship between cognitive functioning and psychopathology in patients with psychiatric disorders: a transdiagnostic network analysis. Psychol. Med..

[bib21] Cheng Z., Moser A.D., Jones M., Kaiser R.H. (2024). Reinforcement learning and working memory in mood disorders: a computational analysis in a developmental transdiagnostic sample. J. Affect. Disord..

[bib22] Collins A.G.E. (2018). The tortoise and the hare: interactions between reinforcement learning and working memory. J. Cogn. Neurosci..

[bib23] Collins A.G.E., Albrecht M.A., Waltz J.A., Gold J.M., Frank M.J. (2017). Interactions among working memory, reinforcement learning, and effort in Value-Based choice: a new paradigm and selective deficits in schizophrenia. Biol. Psychiatry.

[bib24] Collins A.G.E., Brown J.K., Gold J.M., Waltz J.A., Frank M.J. (2014). Working memory contributions to reinforcement learning impairments in schizophrenia. J. Neurosci..

[bib25] Collins A.G.E., Ciullo B., Frank M.J., Badre D. (2017). Working memory load strengthens reward prediction errors. J. Neurosci..

[bib26] Collins A.G.E., Frank M.J. (2012). How much of reinforcement learning is working memory, not reinforcement learning? A behavioral, computational, and neurogenetic analysis. Eur. J. Neurosci..

[bib27] Collins A.G.E., Frank M.J. (2018). Within- and across-trial dynamics of human EEG reveal cooperative interplay between reinforcement learning and working memory. Proc. Natl. Acad. Sci..

[bib28] Copeland W.E., Shanahan L., Costello E.J., Angold A. (2009). Childhood and adolescent psychiatric disorders as predictors of young adult disorders. Arch. Gen. Psychiatry.

[bib29] Cowan N. (2010). The magical mystery four: how is working memory capacity limited, and why?. Curr. Dir. Psychol. Sci..

[bib30] Davidow J.Y., Foerde K., Galván A., Shohamy D. (2016). An upside to reward sensitivity: the hippocampus supports enhanced reinforcement learning in adolescence. Neuron.

[bib31] Davis R., Moray N., Treisman A. (1961). Imitative responses and the rate of gain of information. Q. J. Exp. Psychol..

[bib32] Delevich K., Piekarski D., Wilbrecht L. (2019). Neuroscience: sex hormones at work in the neocortex. Curr. Biol..

[bib33] Delevich K., Thomas A.W., Wilbrecht L. (2018). Adolescence and “Late Blooming” synapses of the prefrontal cortex. Cold Spring Harb. Symp. Quant. Biol..

[bib34] Dickson H., Laurens K.R., Cullen A.E., Hodgins S. (2012). Meta-analyses of cognitive and motor function in youth aged 16 years and younger who subsequently develop schizophrenia. Psychol. Med..

[bib35] Eckstein M.K., Master S.L., Xia L., Dahl R.E., Wilbrecht L., Collins A.G. (2022). The interpretation of computational model parameters depends on the context. eLife.

[bib36] Ferguson H.J., Brunsdon V.E.A., Bradford E.E.F. (2021). The developmental trajectories of executive function from adolescence to old age. Sci. Rep..

[bib37] Fergusson D.M., Horwood L.J., Ridder E.M., Beautrais A.L. (2005). Subthreshold depression in adolescence and mental health outcomes in adulthood. Arch. Gen. Psychiatry.

[bib38] Fontanesi L., Gluth S., Spektor M.S., Rieskamp J. (2019). A reinforcement learning diffusion decision model for value-based decisions. Psychon. Bull. Rev..

[bib39] Forstmann B.U., Ratcliff R., Wagenmakers E.-J. (2016). Sequential sampling models in cognitive neuroscience: advantages, applications, and extensions. Annu. Rev. Psychol..

[bib40] Forstmann B.U., Tittgemeyer M., Wagenmakers E.-J., Derrfuss J., Imperati D., Brown S. (2011). The Speed-Accuracy tradeoff in the elderly brain: a structural Model-Based approach. J. Neurosci..

[bib41] Frank M.J., Gagne C., Nyhus E., Masters S., Wiecki T.V., Cavanagh J.F., Badre D. (2015). fMRI and EEG predictors of dynamic decision parameters during human reinforcement learning. J. Neurosci..

[bib42] Frank M.J., Moustafa A.A., Haughey H.M., Curran T., Hutchison K.E. (2007). Genetic triple dissociation reveals multiple roles for dopamine in reinforcement learning. Proc. Natl. Acad. Sci..

[bib43] Georgiades K., Lewinsohn P.M., Monroe S.M., Seeley J.R. (2006). Major depressive disorder in adolescence: the role of subthreshold symptoms. J. Am. Acad. Child Adolesc. Psychiatry.

[bib44] Goldman-Mellor S.J., Caspi A., Harrington H., Hogan S., Nada-Raja S., Poulton R., Moffitt T.E. (2014). Suicide attempt in young people: a signal for Long-term health care and social needs. JAMA Psychiatry.

[bib45] Gopnik A. (2020). Childhood as a solution to explore–exploit tensions. Philos. Trans. R. Soc. B Biol. Sci..

[bib46] Gur R.C., Calkins M.E., Satterthwaite T.D., Ruparel K., Bilker W.B., Moore T.M., Savitt A.P., Hakonarson H., Gur R.E. (2014). Neurocognitive growth charting in psychosis spectrum youths. JAMA Psychiatry.

[bib47] Halahakoon D.C., Kieslich K., O’Driscoll C., Nair A., Lewis G., Roiser J.P. (2020). Reward-Processing behavior in depressed participants relative to healthy volunteers: a systematic review and Meta-analysis. JAMA Psychiatry.

[bib48] Hauser T.U., Iannaccone R., Walitza S., Brandeis D., Brem S. (2015). Cognitive flexibility in adolescence: neural and behavioral mechanisms of reward prediction error processing in adaptive decision making during development. NeuroImage.

[bib49] Herr N.R., Williams J.W., Jr, Benjamin S., McDuffie J. (2014). Does this patient have generalized anxiety or panic disorder?: the rational clinical examination systematic review. JAMA.

[bib50] Hick W.E. (1952). On the rate of gain of information. Q. J. Exp. Psychol..

[bib51] Hong, S.H., Zou, A.R., Yoo, A.H., & Collins, A. (2024). Episodic memory contributions to working memory-supported reinforcement learning. https://doi.org/10.31234/osf.io/64hxe. 10.1037/xlm0001456 40167563

[bib52] Huizinga M., Dolan C.V., van der Molen M.W. (2006). Age-related change in executive function: developmental trends and a latent variable analysis. Neuropsychologia.

[bib53] Huys Q.J.M., Browning M., Paulus M.P., Frank M.J. (2021). Advances in the computational understanding of mental illness. Neuropsychopharmacology.

[bib54] Huys Q.J.M., Maia T.V., Frank M.J. (2016). Computational psychiatry as a bridge from neuroscience to clinical applications. Nat. Neurosci..

[bib55] Huys Q.J.M., Pizzagalli D.A., Bogdan R., Dayan P. (2013). Mapping anhedonia onto reinforcement learning: a behavioural meta-analysis. Biol. Mood Anxiety Disord..

[bib56] Hyman R. (1953). Stimulus information as a determinant of reaction time. J. Exp. Psychol..

[bib57] Ironside M., Amemori K., McGrath C.L., Pedersen M.L., Kang M.S., Amemori S., Frank M.J., Graybiel A.M., Pizzagalli D.A. (2020). Approach-Avoidance conflict in major depressive disorder: congruent neural findings in humans and nonhuman primates. Biol. Psychiatry.

[bib58] Jeffreys, H. (1961). The Theory of Probability (3rd ed.). OUP Oxford.

[bib59] Jinnin R., Okamoto Y., Takagaki K., Nishiyama Y., Yamamura T., Okamoto Y., Miyake Y., Takebayashi Y., Tanaka K., Sugiura Y., Shimoda H., Kawakami N., Furukawa T.A., Yamawaki S. (2016). Detailed course of depressive symptoms and risk for developing depression in late adolescents with subthreshold depression: a cohort study. Neuropsychiatr. Dis. Treat..

[bib60] Johnson J.G., Cohen P., Kasen S. (2009). Minor depression during adolescence and mental health outcomes during adulthood. Br. J. Psychiatry.

[bib61] Juraska J.M., Willing J. (2017). Pubertal onset as a critical transition for neural development and cognition. Brain Res..

[bib62] Kessler R.C., Avenevoli S., Costello E.J., Georgiades K., Green J.G., Gruber M.J., He J., Koretz D., McLaughlin K.A., Petukhova M., Sampson N.A., Zaslavsky A.M., Merikangas K.R. (2012). Prevalence, persistence, and sociodemographic correlates of DSM-IV disorders in The National comorbidity survey replication adolescent supplement. Arch. Gen. Psychiatry.

[bib63] Kessler R.C., Chiu W.T., Demler O., Walters E.E. (2005). Prevalence, severity, and comorbidity of 12-Month DSM-IV disorders in The National comorbidity survey replication. Arch. Gen. Psychiatry.

[bib64] Larsen B., Luna B. (2018). Adolescence as a neurobiological critical period for the development of higher-order cognition. Neurosci. Biobehav. Rev..

[bib65] Le T.M., Borghi J.A., Kujawa A.J., Klein D.N., Leung H.-C. (2017). Alterations in visual cortical activation and connectivity with prefrontal cortex during working memory updating in major depressive disorder. NeuroImage Clin..

[bib66] Lefebvre G., Lebreton M., Meyniel F., Bourgeois-Gironde S., Palminteri S. (2017). Behavioural and neural characterization of optimistic reinforcement learning. Nat. Hum. Behav..

[bib67] Liddell T.M., Kruschke J.K. (2018). Analyzing ordinal data with metric models: what could possibly go wrong?. J. Exp. Soc. Psychol..

[bib68] MacLeod C., Mathews A., Tata P. (1986). Attentional bias in emotional disorders. J. Abnorm. Psychol..

[bib69] Master S.L., Eckstein M.K., Gotlieb N., Dahl R.E., Wilbrecht L., Collins A.G.E. (2020). Disentangling the systems contributing to changes in learning during adolescence. Dev. Cogn. Neurosci..

[bib70] McDougle S.D., Collins A.G.E. (2021). Modeling the influence of working memory, reinforcement, and action uncertainty on reaction time and choice during instrumental learning. Psychon. Bull. Rev..

[bib71] Mowbray G.H., Rhoades M.V. (1959). On the reduction of choice reaction times with practice. Q. J. Exp. Psychol..

[bib72] Nikolin S., Tan Y.Y., Schwaab A., Moffa A., Loo C.K., Martin D. (2021). An investigation of working memory deficits in depression using the n-back task: a systematic review and meta-analysis. J. Affect. Disord..

[bib73] Noyes B.K., Munoz D.P., Khalid-Khan S., Brietzke E., Booij L. (2022). Is subthreshold depression in adolescence clinically relevant?. J. Affect. Disord..

[bib74] Nussenbaum K., Hartley C.A. (2019). Reinforcement learning across development: what insights can we draw from a decade of research?. Dev. Cogn. Neurosci..

[bib75] Oberauer K., Lewandowsky S., Awh E., Brown G.D.A., Conway A., Cowan N., Donkin C., Farrell S., Hitch G.J., Hurlstone M.J., Ma W.J., Morey C.C., Nee D.E., Schweppe J., Vergauwe E., Ward G. (2018). Benchmarks for models of short-term and working memory. Psychol. Bull..

[bib76] Palminteri S., Kilford E.J., Coricelli G., Blakemore S.-J. (2016). The computational development of reinforcement learning during adolescence. PLOS Comput. Biol..

[bib77] Paus T., Keshavan M., Giedd J.N. (2008). Why do many psychiatric disorders emerge during adolescence?. Nat. Rev. Neurosci..

[bib78] Pedersen M.L., Frank M.J., Biele G. (2017). The drift diffusion model as the choice rule in reinforcement learning. Psychon. Bull. Rev..

[bib79] Pedersen M.L., Ironside M., Amemori K., McGrath C.L., Kang M.S., Graybiel A.M., Pizzagalli D.A., Frank M.J. (2021). Computational phenotyping of brain-behavior dynamics underlying approach-avoidance conflict in major depressive disorder. PLOS Comput. Biol..

[bib80] Pedregosa F., Varoquaux G., Gramfort A., Michel V., Thirion B., Grisel O., Blondel M., Prettenhofer P., Weiss R., Dubourg V., Vanderplas J., Passos A., Cournapeau D., Brucher M., Perrot M., Duchesnay É. (2011). Scikit-learn: machine learning in python. J. Mach. Learn. Res..

[bib81] Piekarski D.J., Johnson C.M., Boivin J.R., Thomas A.W., Lin W.C., Delevich K., M. Galarce E., Wilbrecht L. (2017). Does puberty mark a transition in sensitive periods for plasticity in the associative neocortex?. Brain Res..

[bib82] Pike A.C., Robinson O.J. (2022). Reinforcement learning in patients with mood and anxiety disorders vs control individuals: a systematic review and Meta-analysis. JAMA Psychiatry.

[bib83] Pine D.S., Cohen E., Cohen P., Brook J. (1999). Adolescent depressive symptoms as predictors of adult depression: moodiness or mood disorder?. Am. J. Psychiatry.

[bib84] Pine D.S., Cohen P., Gurley D., Brook J., Ma Y. (1998). The risk for Early-Adulthood anxiety and depressive disorders in adolescents with anxiety and depressive disorders. Arch. Gen. Psychiatry.

[bib85] Proctor R.W., Schneider D.W. (2018). Hick’s law for choice reaction time: a review. Q. J. Exp. Psychol..

[bib86] Rabbitt P.M.A. (1968). Repetition effects and signal classification strategies in serial Choice-Response tasks. Q. J. Exp. Psychol..

[bib87] Ratcliff R. (1978). A theory of memory retrieval. Psychol. Rev..

[bib88] Ratcliff R., Childers R. (2015). Individual differences and fitting methods for the two-choice diffusion model of decision making. Decision.

[bib89] Ratcliff R., Love J., Thompson C.A., Opfer J.E. (2012). Children are not like older adults: a diffusion model analysis of developmental changes in speeded responses. Child Dev..

[bib90] Remington R.J. (1969). Analysis of sequential effects on choice reaction times. J. Exp. Psychol..

[bib91] Rescorla R.A., Wagner A.R. (1972). A theory of pavlovian conditioning: variations in the effectiveness of reinforcement and non-reinforcement. Class. Cond. Curr. Res. Theory.

[bib92] Rodwell L., Romaniuk H., Nilsen W., Carlin J.B., Lee K.J., Patton G.C. (2018). Adolescent mental health and behavioural predictors of being NEET: a prospective study of young adults not in employment, education, or training. Psychol. Med..

[bib93] Rose E.J., Ebmeier K.P. (2006). Pattern of impaired working memory during major depression. J. Affect. Disord..

[bib94] Sawyer S.M., Azzopardi P.S., Wickremarathne D., Patton G.C. (2018). The age of adolescence. Lancet Child Adolesc. Health.

[bib95] Schneider D.W., Anderson J.R. (2011). A memory-based model of Hick’s law. Cogn. Psychol..

[bib96] Schreuders E., Braams B.R., Blankenstein N.E., Peper J.S., Güroğlu B., Crone E.A. (2018). Contributions of reward sensitivity to ventral striatum activity across adolescence and early adulthood. Child Dev..

[bib97] Schumacher A., Campisi S.C., Khalfan A.F., Merriman K., Williams T.S., Korczak D.J. (2024). Cognitive functioning in children and adolescents with depression: a systematic review and meta-analysis. Eur. Neuropsychopharmacol..

[bib98] Senta, J., Bishop, S., & Collins, A.G. (2025). Dual process impairments in reinforcement learning and working memory systems underlie learning deficits in physiological anxiety (p. 2025.02.14.638024). bioRxiv. https://doi.org/10.1101/2025.02.14.638024. 10.1371/journal.pcbi.1012872 PMC12500139 41004539

[bib99] Shahar N., Hauser T.U., Moutoussis M., Moran R., Keramati M., Consortium N., Dolan R.J. (2019). Improving the reliability of model-based decision-making estimates in the two-stage decision task with reaction-times and drift-diffusion modeling. PLOS Comput. Biol..

[bib100] Shannon C.E. (1948).

[bib101] Solmi M., Radua J., Olivola M., Croce E., Soardo L., Salazar de Pablo G., Il Shin J., Kirkbride J.B., Jones P., Kim J.H., Kim J.Y., Carvalho A.F., Seeman M.V., Correll C.U., Fusar-Poli P. (2022). Age at onset of mental disorders worldwide: Large-scale meta-analysis of 192 epidemiological studies. Mol. Psychiatry.

[bib102] Spitzer R.L., Kroenke K., Williams J.B.W., Löwe B. (2006). A brief measure for assessing generalized anxiety disorder: the GAD-7. Arch. Intern. Med..

[bib103] Steinberg L. (2005). Cognitive and affective development in adolescence. Trends Cogn. Sci..

[bib104] Sumner, E., Li, A., Perfors, A., Hayes, B., Navarro, D., & Sarnecka, B. (2019). The Exploration Advantage: Children’s instinct to explore allows them to find information that adults miss. OSF. https://doi.org/10.31234/osf.io/h437v.

[bib105] Sutton, R.S., & Barto, A.G. (2018). Reinforcement Learning: An Introduction (2nd ed.). The MIT Press. 〈https://web.stanford.edu/class/psych209/Readings/SuttonBartoIPRLBook2ndEd.pdf〉.

[bib106] Tervo-Clemmens B., Calabro F.J., Parr A.C., Fedor J., Foran W., Luna B. (2023). A canonical trajectory of executive function maturation from adolescence to adulthood. Nat. Commun..

[bib107] Theisen M., Lerche V., von Krause M., Voss A. (2021). Age differences in diffusion model parameters: a meta-analysis. Psychol. Res..

[bib108] Tichelaar J.G., Hezemans F., Bloem B.R., Helmich R.C., Cools R. (2025). Neural reinforcement learning signals predict recovery from impulse control disorder symptoms in Parkinson’s disease. Biol. Psychiatry.

[bib109] Wagenmakers E.-J., Lodewyckx T., Kuriyal H., Grasman R. (2010). Bayesian hypothesis testing for psychologists: a tutorial on the Savage–Dickey method. Cogn. Psychol..

[bib110] Wagenmakers E.-J., Love J., Marsman M., Jamil T., Ly A., Verhagen J., Selker R., Gronau Q.F., Dropmann D., Boutin B., Meerhoff F., Knight P., Raj A., van Kesteren E.-J., van Doorn J., Šmíra M., Epskamp S., Etz A., Matzke D., Morey R.D. (2018). Bayesian inference for psychology. Part II: example applications with JASP. Psychon. Bull. Rev..

[bib111] Walker D.M., Bell M.R., Flores C., Gulley J.M., Willing J., Paul M.J. (2017). Adolescence and reward: making sense of neural and behavioral changes amid the chaos. J. Neurosci..

[bib112] Waltmann M., Herzog N., Reiter A.M.F., Villringer A., Horstmann A., Deserno L. (2023). Diminished reinforcement sensitivity in adolescence is associated with enhanced response switching and reduced coding of choice probability in the medial frontal pole. Dev. Cogn. Neurosci..

[bib113] Wiecki T.V., Poland J., Frank M.J. (2015). Model-Based cognitive neuroscience approaches to computational psychiatry: clustering and classification. Clin. Psychol. Sci..

[bib114] Wilbrecht L., Davidow J.Y. (2024). Goal-directed learning in adolescence: neurocognitive development and contextual influences. Nat. Rev. Neurosci..

[bib115] Wilson R.C., Geana A., White J.M., Ludvig E.A., Cohen J.D. (2014). Humans use directed and random exploration to solve the explore–exploit dilemma. J. Exp. Psychol. Gen..

[bib116] Yoo A.H., Collins A.G.E. (2022). How working memory and reinforcement learning are intertwined: a cognitive, neural, and computational perspective. J. Cogn. Neurosci..

[bib117] Zhu Y., Womer F.Y., Leng H., Chang M., Yin Z., Wei Y., Zhou Q., Fu S., Deng X., Lv J., Song Y., Ma Y., Sun X., Bao J., Wei S., Jiang X., Tan S., Tang Y., Wang F. (2019). The relationship between cognitive dysfunction and symptom dimensions across schizophrenia, bipolar disorder, and major depressive disorder. Front. Psychiatry.

