# Supplementary material for: Linking reinforcement learning, working memory, and choice dynamics to age and symptoms of anxiety and depression in adolescence
Source: Dev Cogn Neurosci. 2025 Oct 8;76:101626. doi: 10.1016/j.dcn.2025.101626 (PMC12549770; doi:10.1016/j.dcn.2025.101626)
Supplement: Table S1 — Supplementary material [file mmc1.docx]

**Supplementary materials**


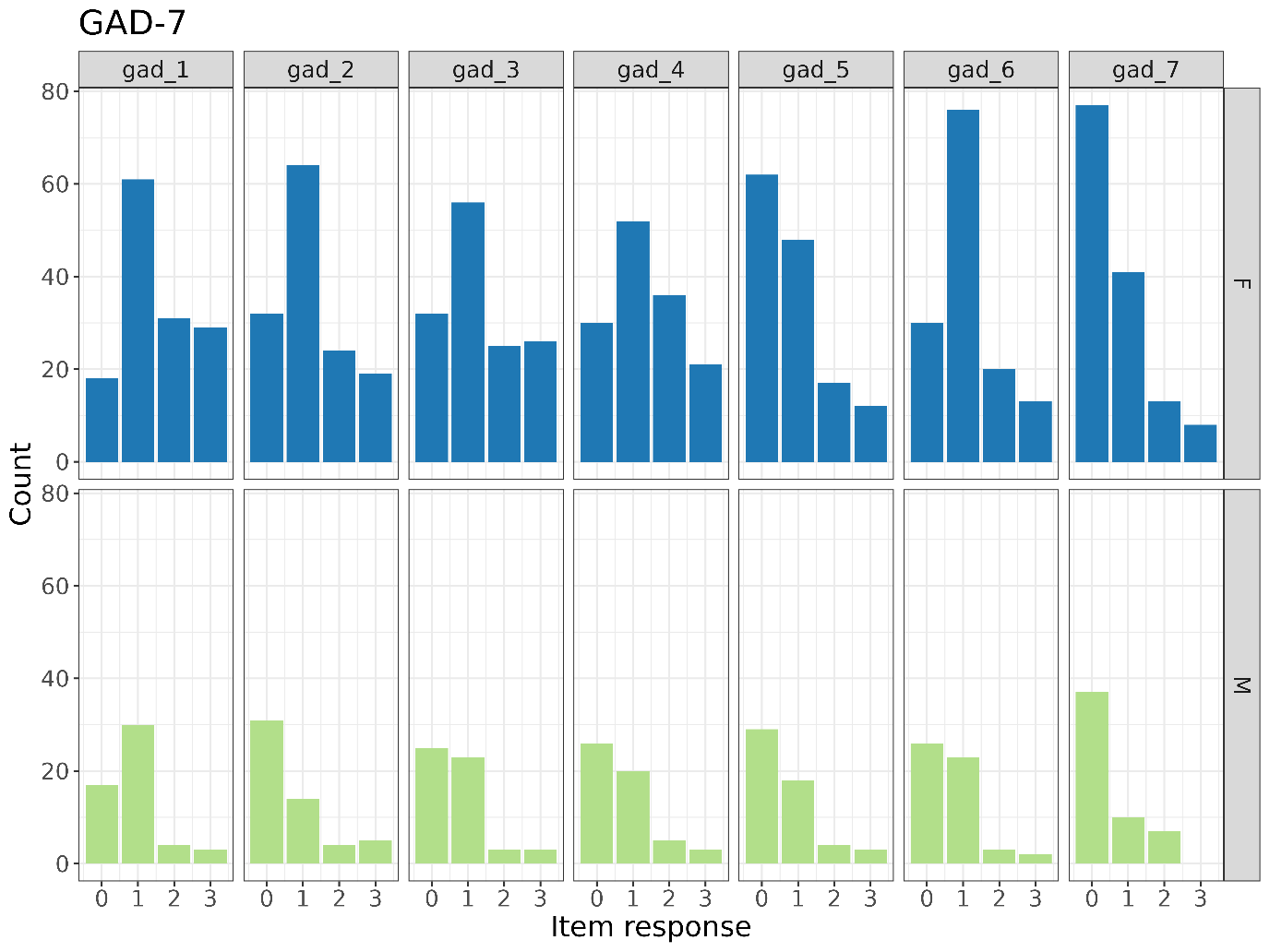


**Fig. S1:** Responses on each GAD-7 item. The questionnaire asks participants how often they have been bothered by the following problems over the past two weeks. gad_1, “Feeling nervous, anxious, or on edge”; gad_2, “Not being able to stop or control worrying”; gad_3, “Worrying too much about different things”; gad_4, “Trouble relaxing”; gad_5, “Being so restless that it is hard to sit still”; gad_6, “Becoming easily annoyed or irritable”; gad_7, “Feeling afraid, as if something awful might happen”; 0, “Not at all”; 1, “Several days”; 2, “More than half the days”; 3, “Nearly every day”; F, females; M, males.

**
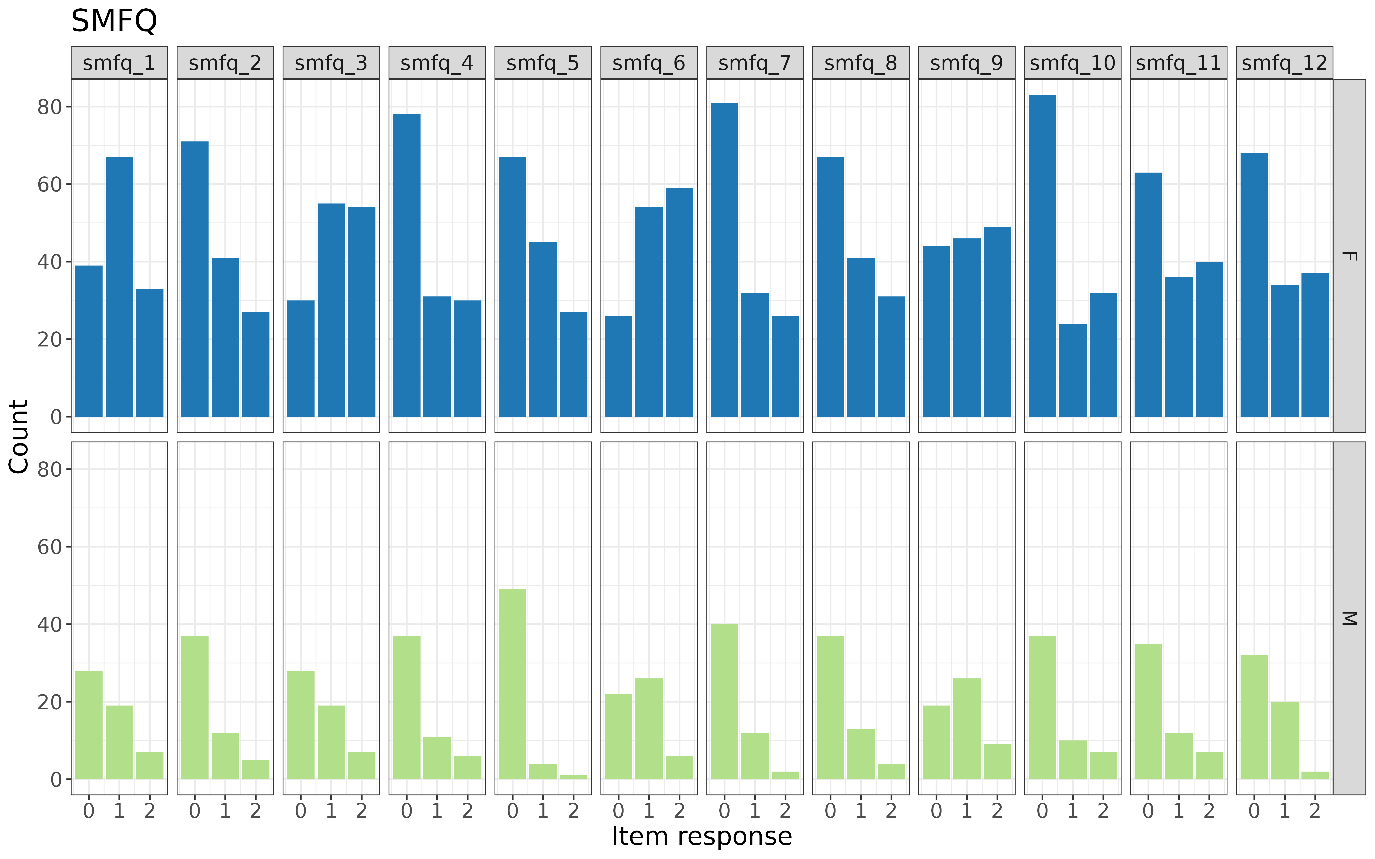
**

**Fig. S2:** Responses on each SMFQ item. The questionnaire prompts participants to respond how much they have felt or acted in the following ways in the past two weeks. smfq_1, “I felt miserable or unhappy”; smfq_2, “I didn’t enjoy anything at all”; smfq_3, “I felt so tired I just sat around and did nothing”; smfq_4, “I felt I was no good any more”; smfq_5, “I cried a lot”; smfq_6, “I found it hard to think properly or concentrate”; smfq_7, “I hated myself”; smfq_8, “I was a bad person”; smfq_9, “I felt lonely”; smfq_10, “I thought nobody really loved me”; smfq_11, “I thought I could never be as good as other kids”; smfq_12, “I did everything wrong”. 0, “not true”; 1, “Sometimes”; 2, “True”; F, females; M, males.

**S1. Model comparisons**

We tested five candidate models that are fit to both choice and reaction time data, with four models based on McDougle & Collins (2021) and the last model replacing the positive learning bias with a negative learning rate. The models are variations of the RLWM-LBA model in the manuscript, referred to as the $\pi_{H}$ model in the present section. A detailed description of the model is presented in *2.4. Computational model*. All candidate models were applied to behavioral data from the RLWM task with a hierarchical approach using Maximum a posteriori estimation.

*S1.1* $\pi$ *model*

The $\pi$ model differs from the $\pi_{H}$ model by not incorporating uncertainty into the evidence accumulation rate. The model assumes a nonlinear relationship between latent action weights, the output of the RLWM module, and accumulation rates. The weights of each action scale the drift rate of their accompanying accumulator, in which each drift rate mean parameter $v_{i}$ is multiplied by the associated weight $\pi_{i}$ (referred to as $P(a|s)$ in manuscript) of each action $a$ on trial $t$:

$v_{a,t}=\eta\pi_{a,t}$ **(1)**

in which $\eta$ is a scaling parameter.

*S1.2* $\pi_{H}$ *model*

The next model assumes that drift rates vary as a function of the estimated weight of each action in the current state, like the model above, and prior uncertainty over actions. See *2.4. Computational model* for a description of the model.

*S1.3* $Q$ *model*

In the $Q$ model, the drift rates are scaled linearly with the latent variables from the separate RL ($Q_{RL}$) and WM ($Q_{WM}$) modules. While the RL and WM modules are weighted differentially, as shown in eq. 6 in the manuscript, there is no nonlinear transformation of latent variables with the softmax function. The mean accumulation rate for each accumulator, $v_{a}$, is proportional to the weighted $Q_{RL}$ and $Q_{WM}$ quantities for each action ($V_{a}$) on trial $t$:

$v_{a,t}=\eta V_{a,t}$ **(2)**

*S1.4* $\pi_{H}RL$ *model*

The $\pi_{H}RL$ model is identical to the $\pi_{H}$ model but has no WM module and thus only a single action policy is learned. The model is included to test the utility of including a WM module in the learning process.

*S1.5* $\alpha\text{-}$*model*

Previous studies using RL have differentiated between learning from positive and negative feedback by including separate learning rates (e.g. Frank et al., 2007; Hauser et al., 2015; Lefebvre et al., 2017). Therefore, we included a model in which the learning rate $\alpha$ and *bias* parameters of the $\pi_{H}$ model are replaced by learning rates for positive, $\alpha\text{+}$, and negative, $\alpha\text{-}$, feedback.

*S1.6 Results*

The strongest model in model comparison was the $\pi_{H}$ model, as shown by the log likelihood, Bayesian Information Criteria (BIC), and Akaike Information Criteria (AIC). We note that the AIC is often used in favor of the BIC for model comparison with the RLWM model, as the BIC has been shown to overpenalize model complexity when generating data from the RLWM model (Collins & Frank, 2012). This may be contributing to the small difference in BIC values between the $\pi_{H}$ and $\pi_{H}RL$ models, in which the latter is fitting fewer parameters. The results are shown in table S1.

| **Model Comparisons** | | | |
| --- | --- | --- | --- |
| **Model** | **LogLik** | **AIC** | **BIC** |
| $\pi$ | −44,717.41 | 92,892.81 | 109,308.89 |
| $\pi_{H}$ | −42,458.22 | 88,374.44 | 104,790.52 |
| $\pi_{H}RL$ | −46,333.79 | 94,827.59 | 105,081.70 |
| $\alpha\text{-}$ | −43,328.53 | 90,115.06 | 106,531.13 |
| $Q$ | −51,755.65 | 106,969.29 | 123,385.37 |

**Table S1:** Model comparisons between candidate models. LogLik, log likelihood; AIC, Akaike Information Criteria; BIC, Bayesian Information Criteria.

**
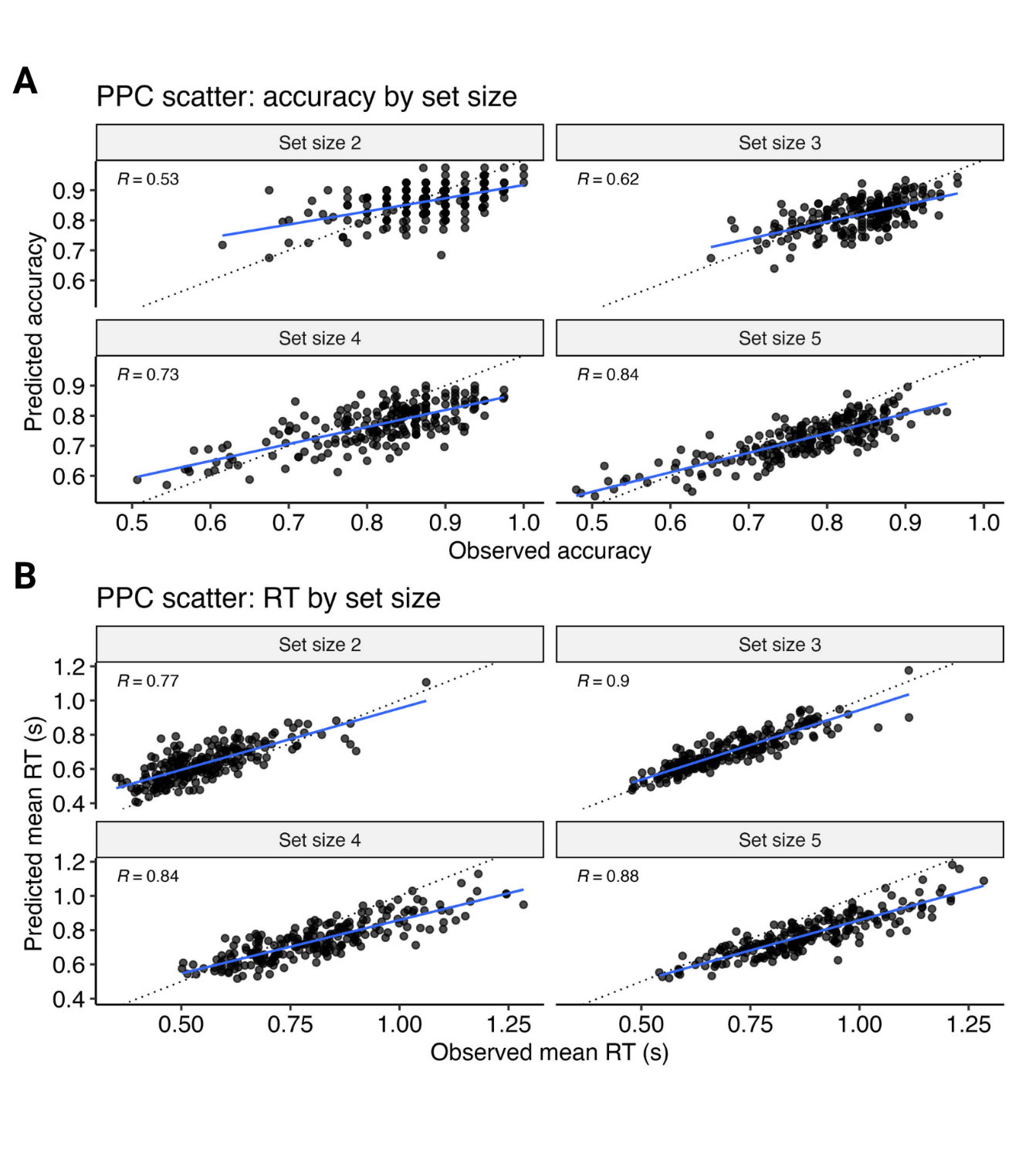
**

**Fig. S3:** Correlations between observed and RLWM-LBA predicted results split between set sizes for A) accuracy and B) reaction times.


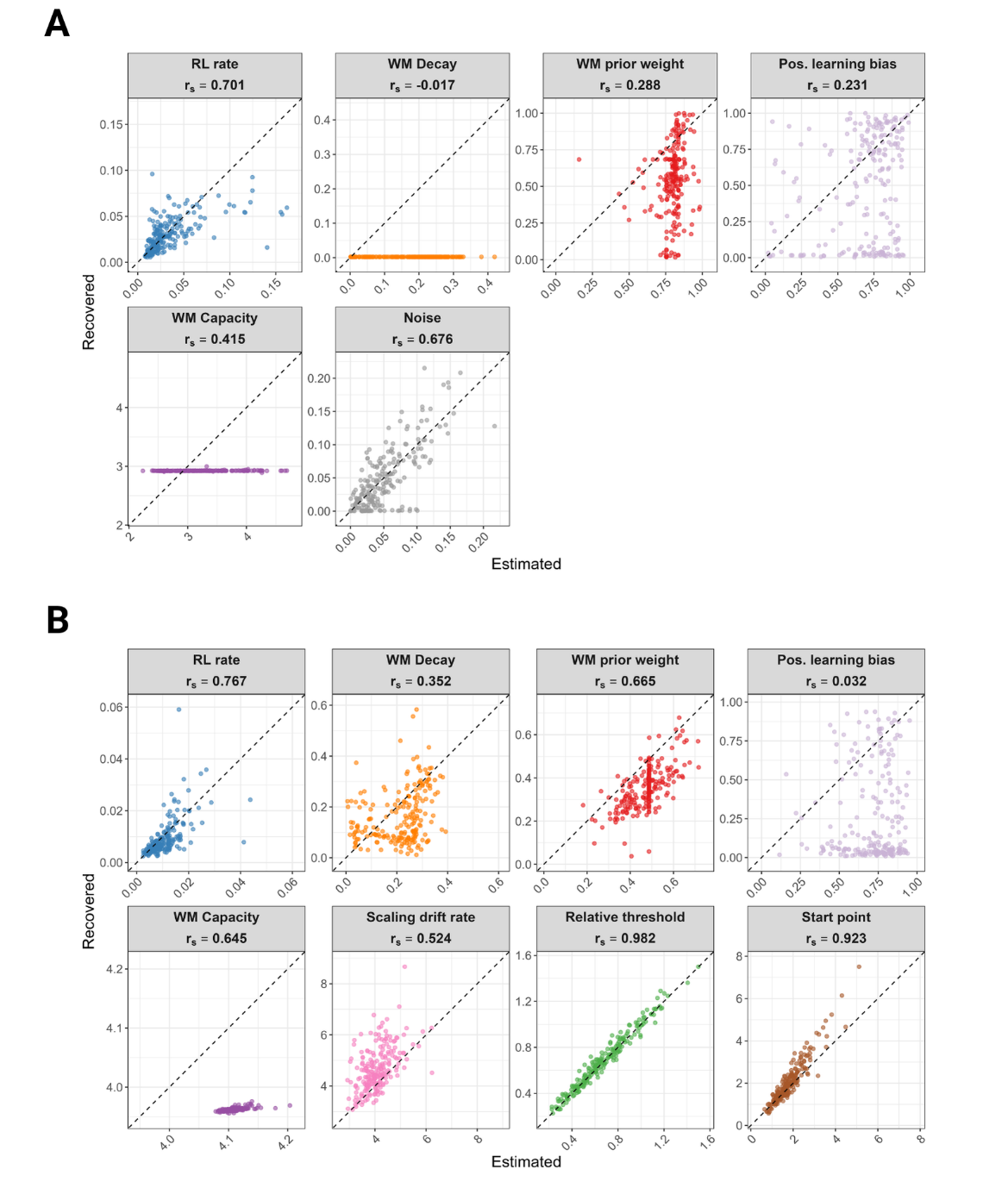


**Fig. S4:** Parameter recovery analyses for the A) RLWM and B) RLWM-LBA models. The RLWM model was similar to previous studies (Collins & Frank, 2012; Master et al., 2020), in which the overall choice policy is defined as a mixture of policies from the WM and RL modules, according to the mixing value $W$. Like the RLWM-LBA, the RLWM model was approximated using maximum a posteriori (MAP) estimation and priors for shared parameters were the same between the models (see table 1 in manuscript). The undirected noise parameter $\epsilon$ is included in the RLWM (equation not shown) to account for “slips” of action in which individuals choose randomly, possible due to lapses in attention.


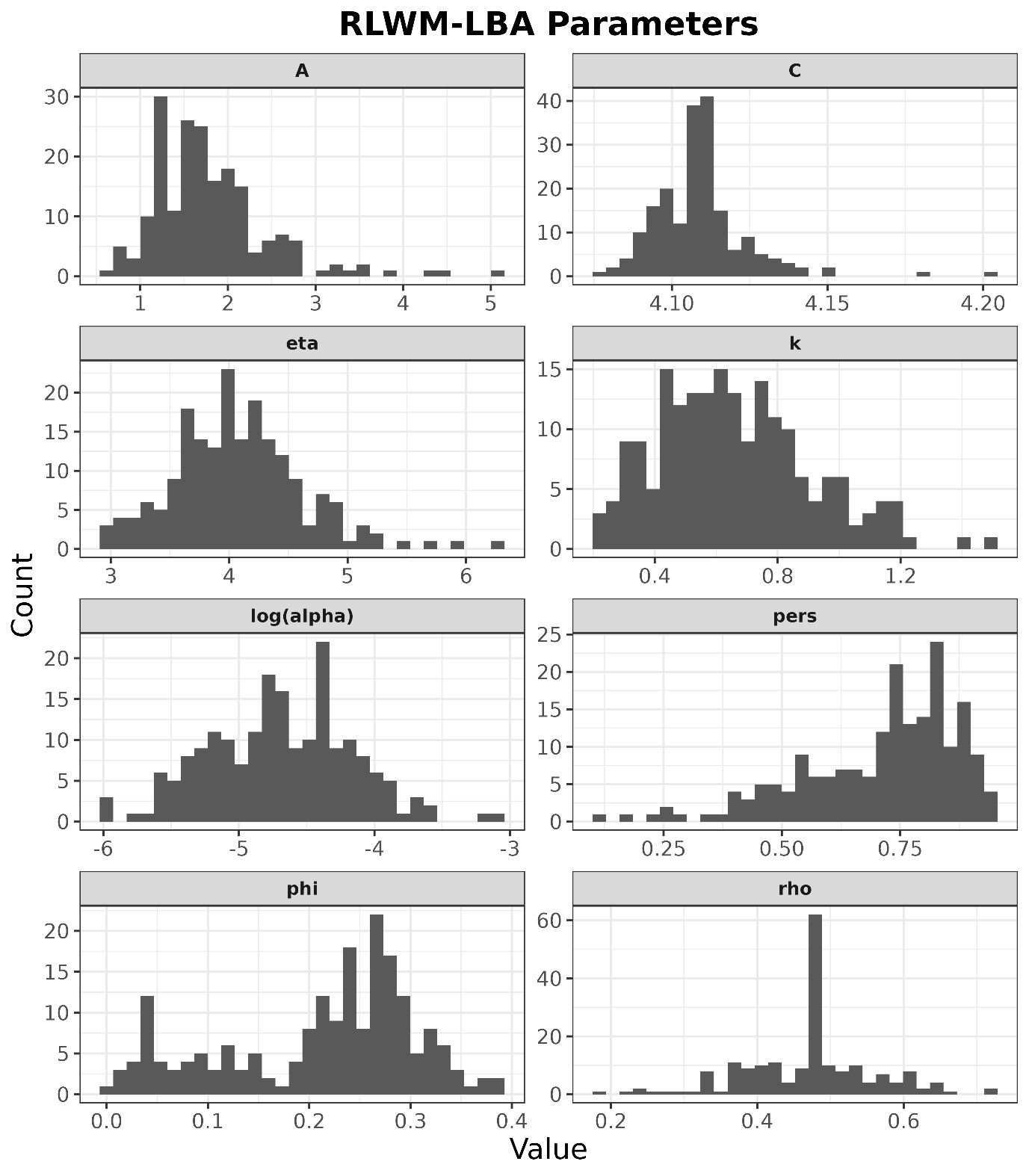


**Fig. S5**: Distributions of the RLWM-LBA parameters. Alpha, RL rate; Phi, WM Decay; Rho, WM prior weight; Pers, positive learning bias; C, WM capacity; Eta, scaling drift rate; k, relative threshold; A, start point variability.

**
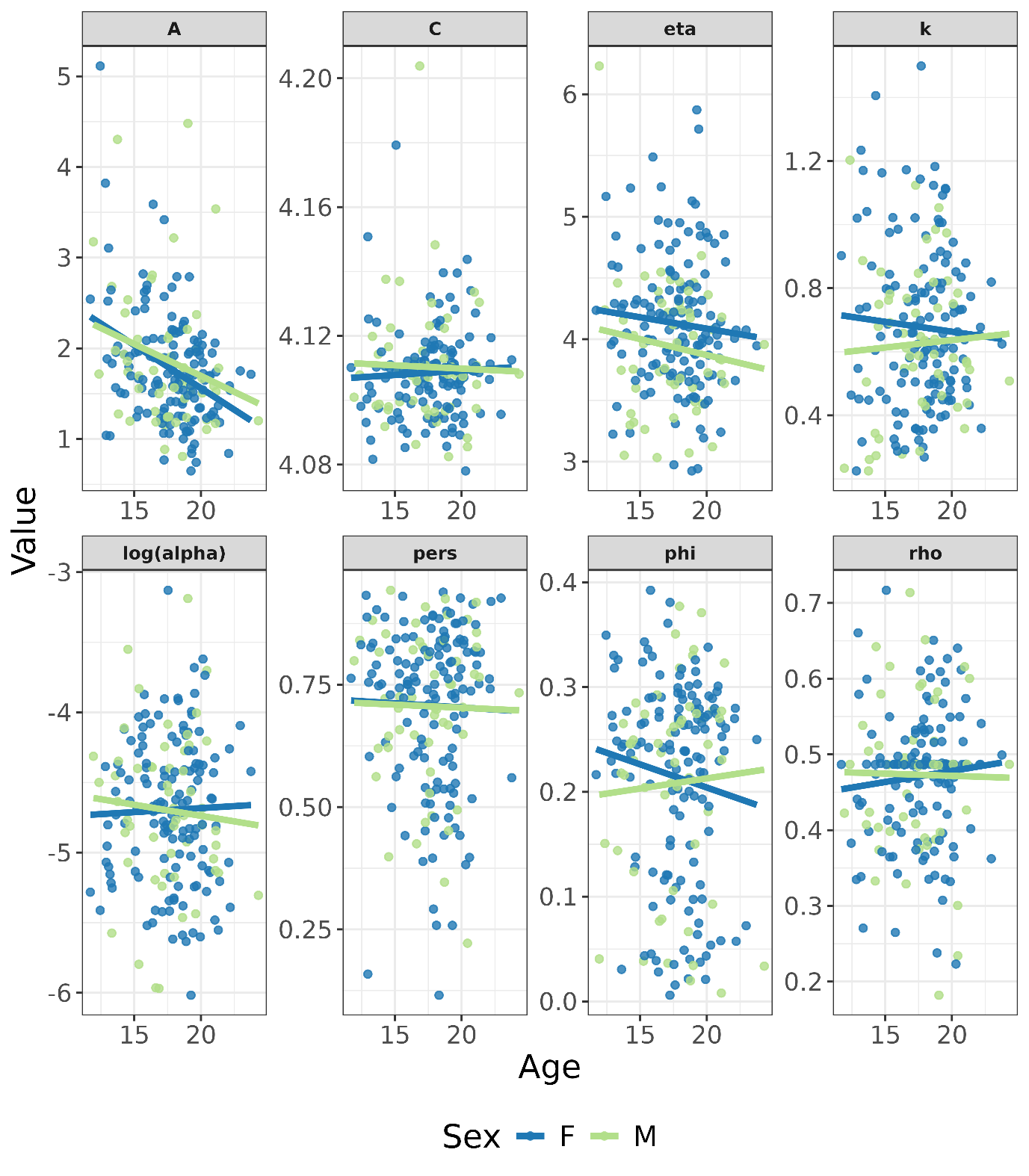
Fig. S6:** Sex-dependent linear relations between age and RLWM-LBA parameter values.


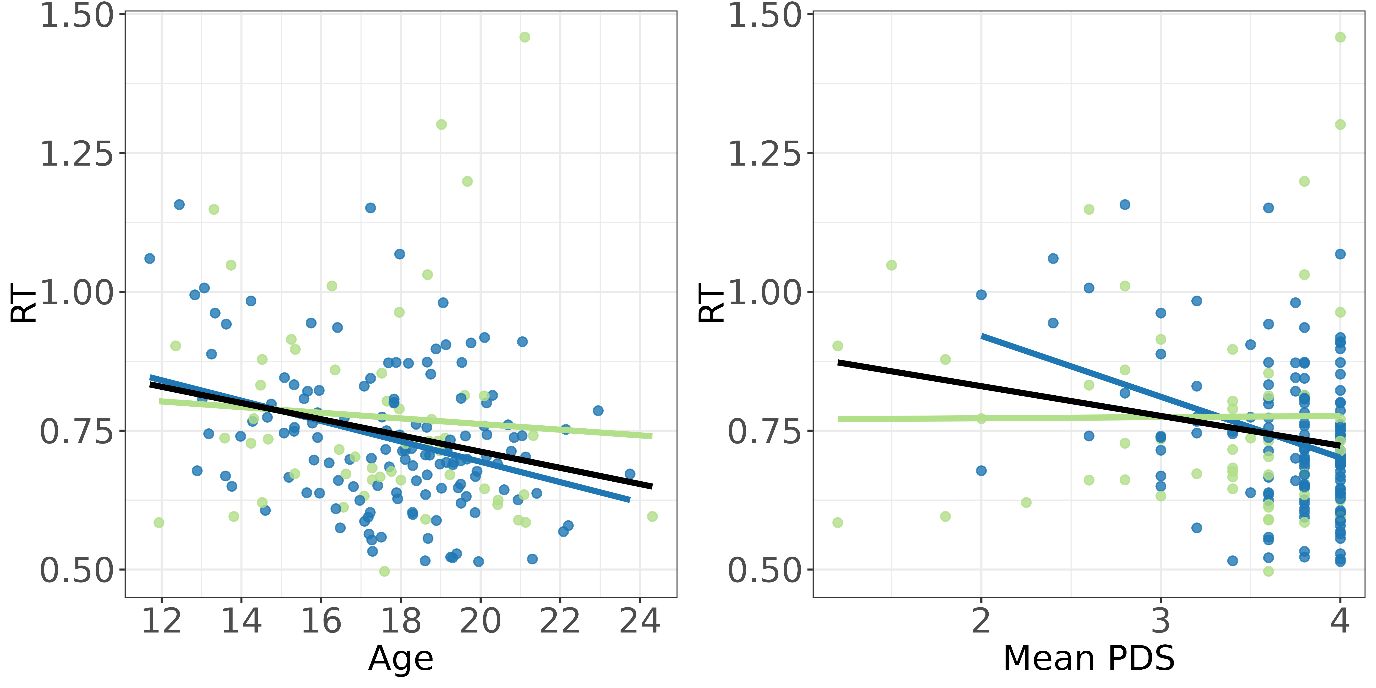


**Fig. S7:** Sex-dependent linear relations between age and reaction time (RT), and mean PDS score and RT. Reaction time is calculated as mean reaction time on correct trials across all set sizes. Females are shown in blue, males in green. The black line is the predicted relationship from a linear model of the form RT ~ X*sex, where X is either age or mean PDS score.


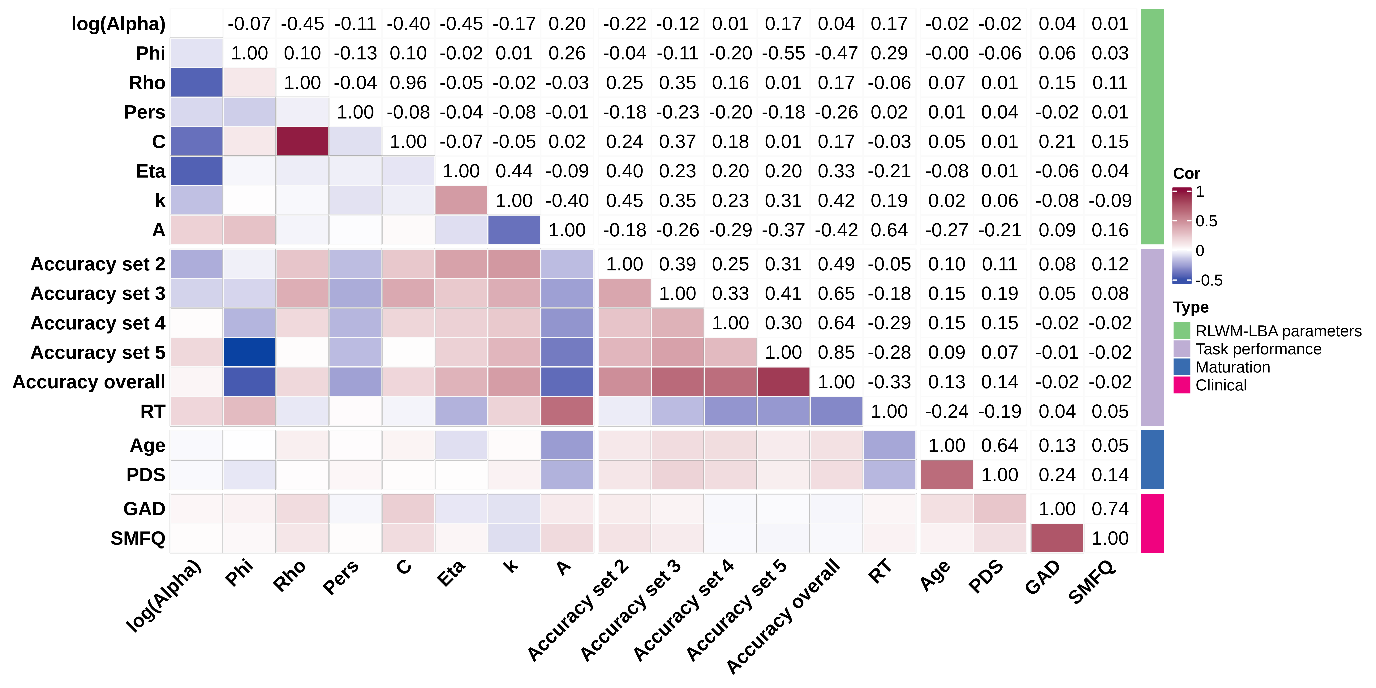


**Fig. S8:** Spearman’s rank correlation matrix showing associations between model parameters, task performance, age, PDS, sum score on the GAD-7 and SMFQ. log(Alpha), log-transformed RL rate; Phi, WM Decay; Rho, WM prior weight; Pers, positive learning bias; C, WM capacity; Eta, scaling drift rate; k, relative threshold; A, start point variability; RT, reaction time on correct trials.

**
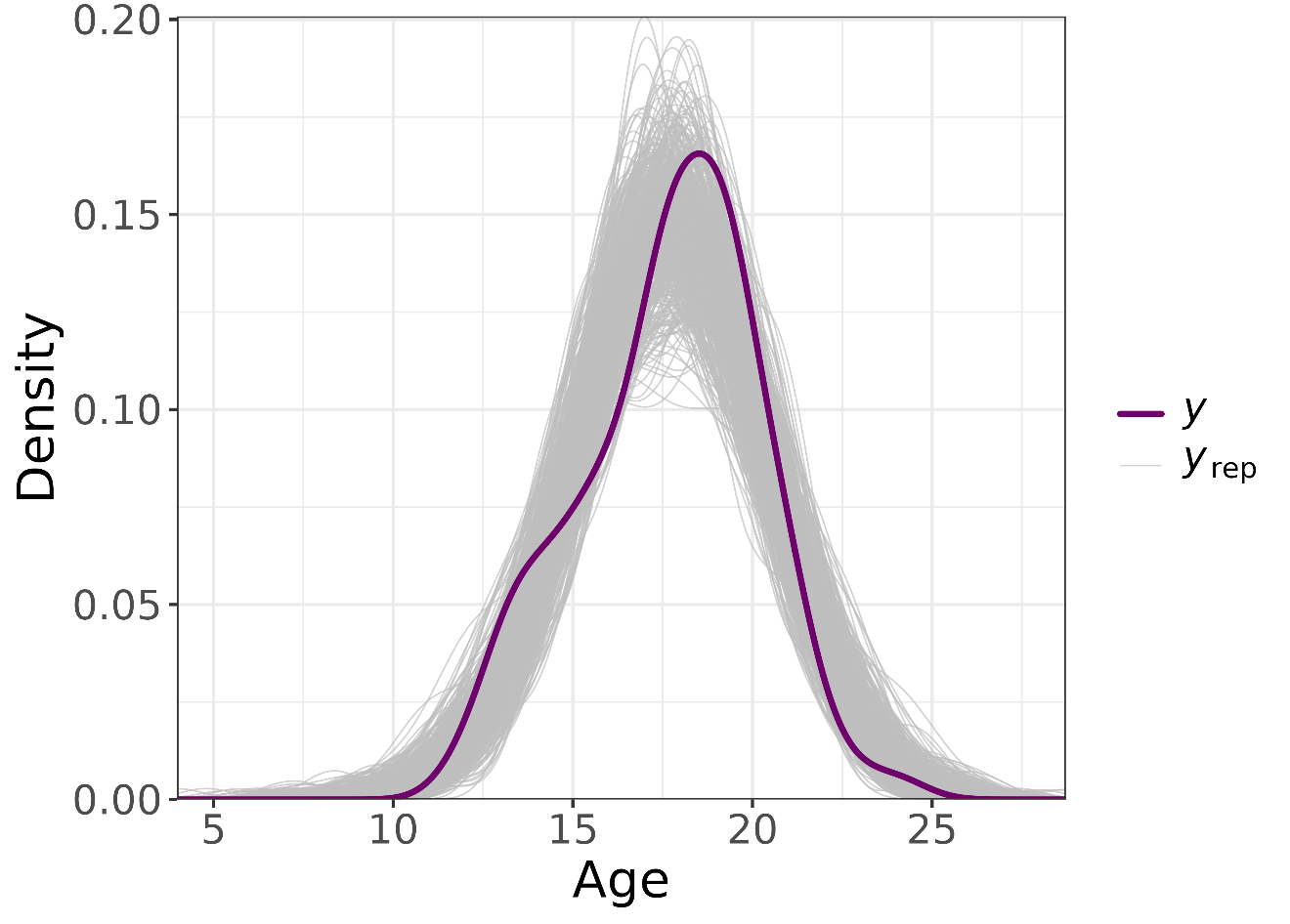
Fig. S9:** Posterior predictive check for the Bayesian regression model with age and model parameters. Model predicted age distribution from 500 samples is shown in light grey and observed age distribution is shown in purple.


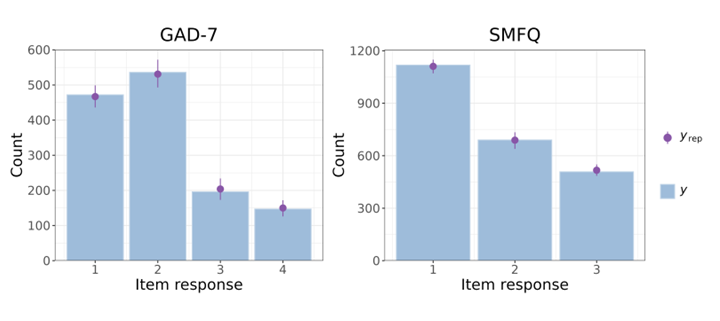
**Fig. S10:** Posterior predictive check for the Bayesian ordinal regression models on GAD-7/SMFQ item response with model parameters, age, and PDS average score as independent variables. Posterior predictive checks were made with 500 random samples from the posterior distribution from (A) the GAD-7 model and (B) the SMFQ model. Predicted response count is shown in purple and observed response count is shown in blue.


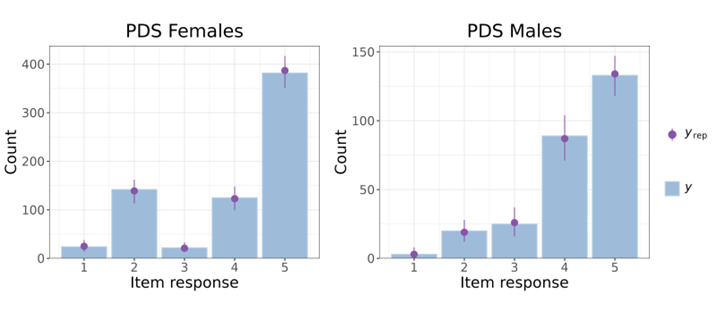
**Fig. S11:** Posterior predictive check for the Bayesian ordinal regression models on PDS item response with model parameters as independent variables, ran separately for males and females. Posterior predictive checks were made with 500 random samples from the posterior distribution from the PDS models. Predicted response count is shown in purple and observed response count is shown in blue.


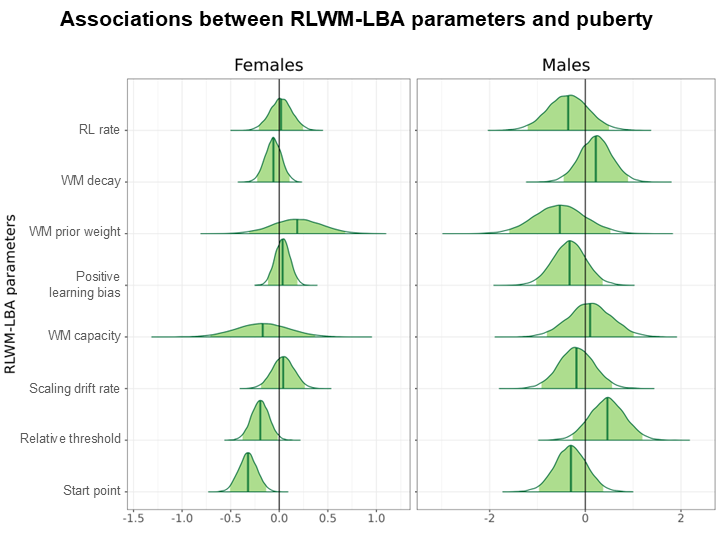
**Fig. S12:** Posterior distributions for the associations between RLWM-LBA parameters and PDS item responses. Green shade in the distributions represent 95% credible interval and vertical green lines in the distributions represent the mean estimate for each association. Females and males responded to different items on the PDS scale, so regression models were run separately. Model output is reported in table S2-3.


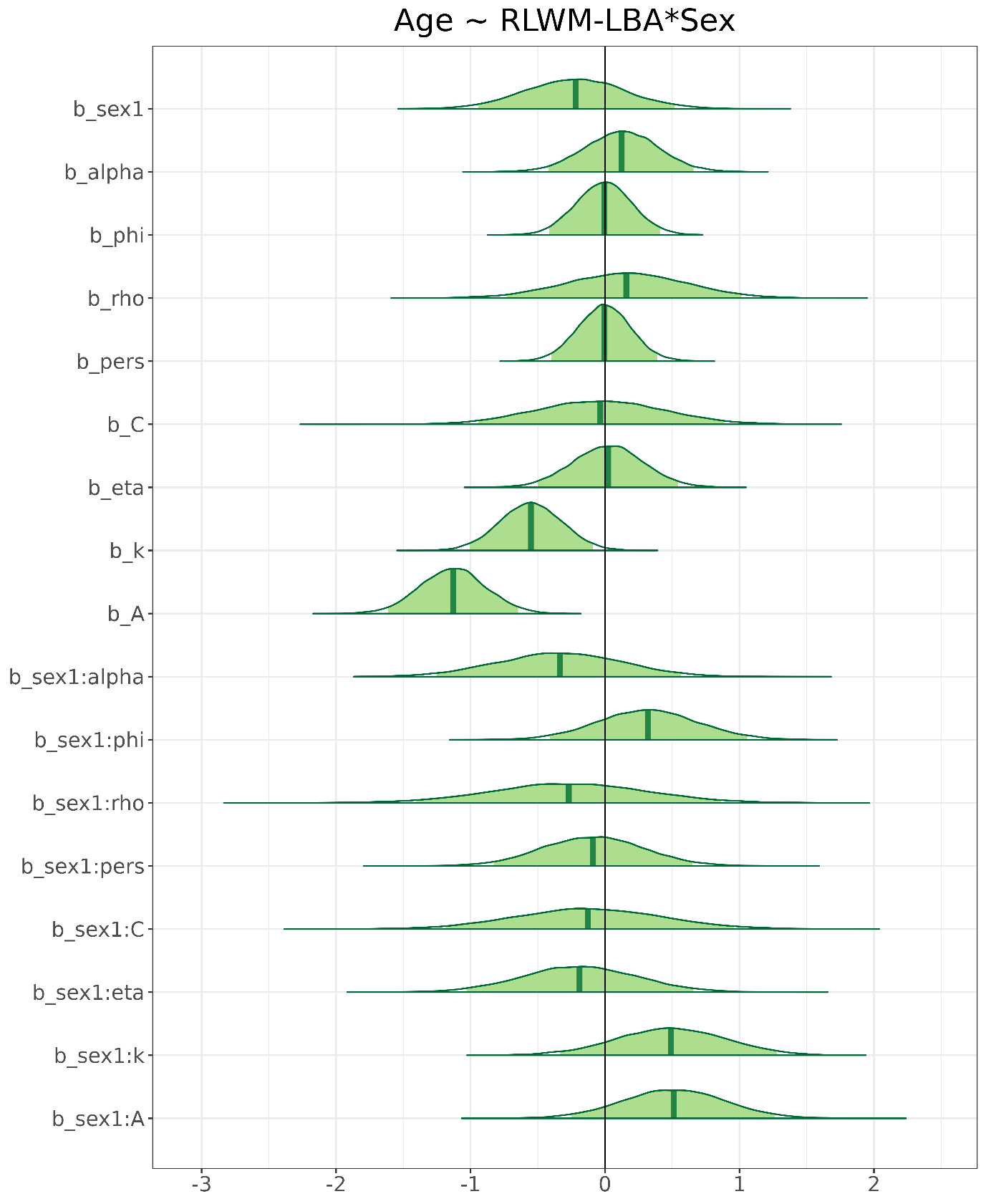
**Fig. S13:** Posterior distributions from the age model with sex (female = 0, male = 1) as interaction with each RLWM-LBA parameter, and association with age (Fig. 6). Model output is shown in table S4. Alpha, RL rate; Phi, WM Decay; Rho, WM prior weight; Pers, positive learning bias; C, WM capacity; Eta, scaling drift rate; k, relative threshold; A, start point variability.


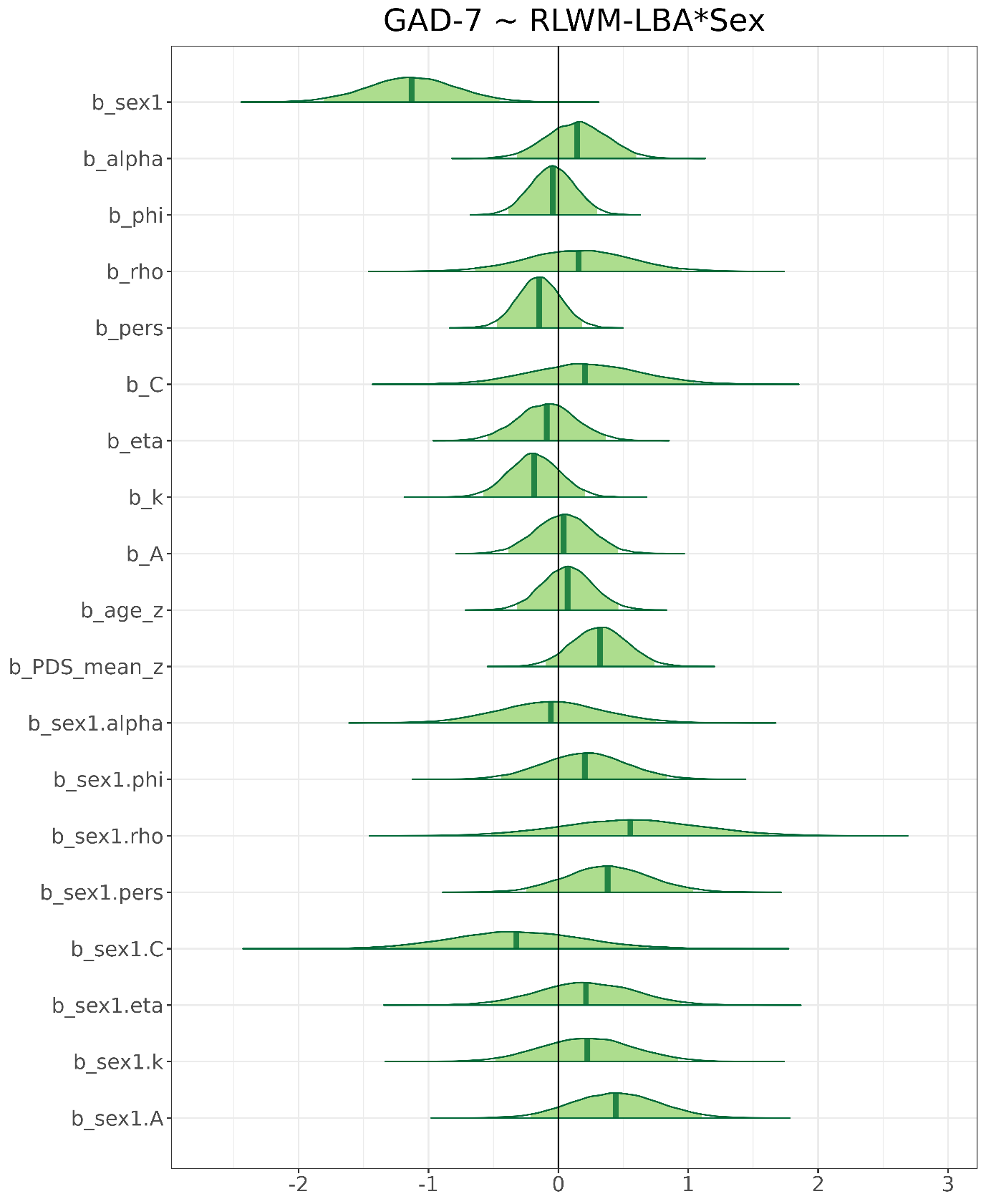


**Fig. S14:** Posterior distributions for all associations from the GAD-7 model (Fig. 7) with sex (female = 1, male = 0) as interaction with each RLWM-LBA parameter, and covariates age and pubertal status (PDS). Model output and BFs are shown in table S5. Alpha, RL rate; Phi, WM Decay; Rho, WM prior weight; Pers, positive learning bias; C, WM capacity; Eta, scaling drift rate; k, relative threshold; A, start point variability.


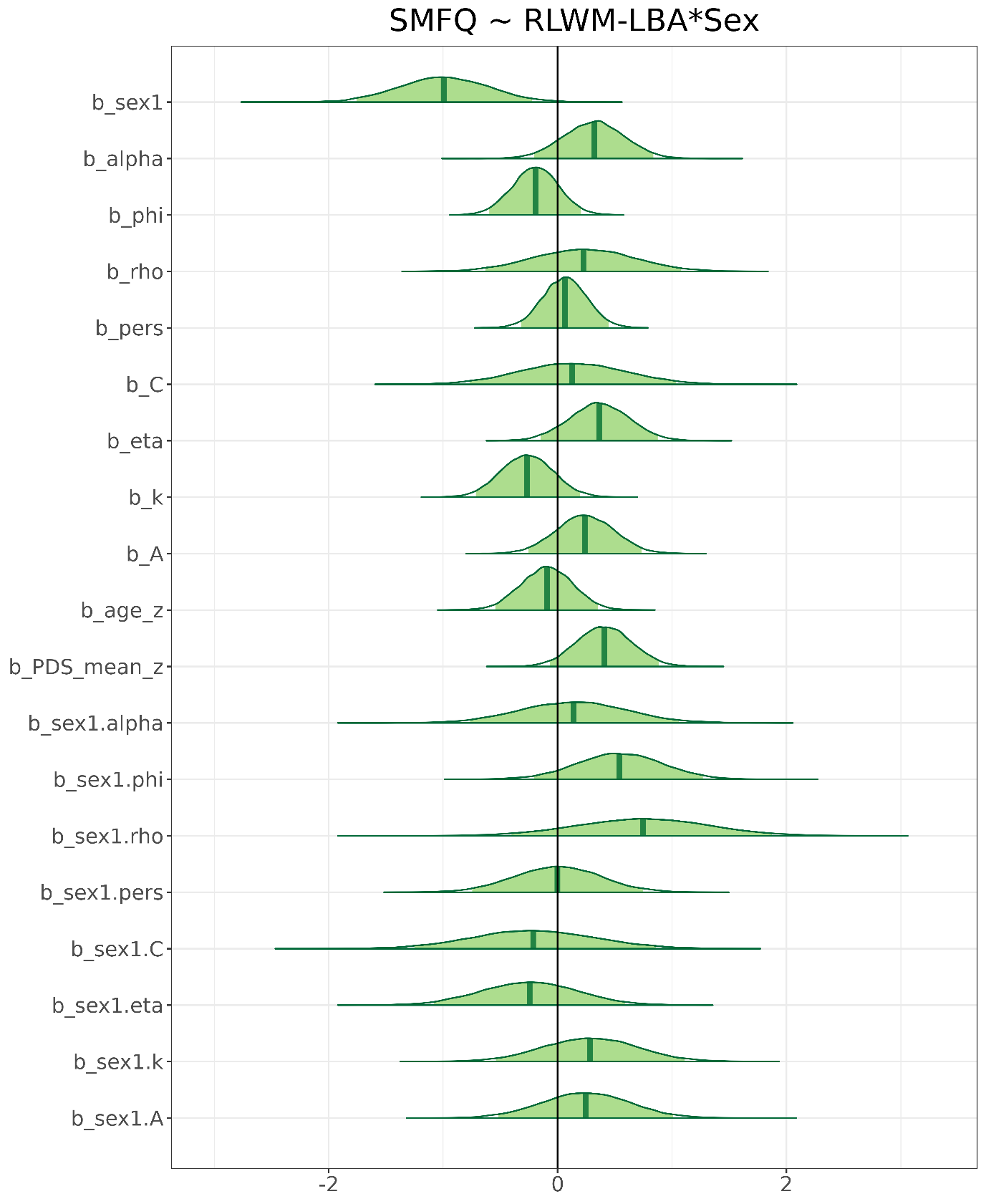
**Fig. S15:** Posterior distributions for all associations from the SMFQ model (Fig. 7) with sex (female = 1, male = 0) as interaction with each RLWM-LBA parameter, and covariates age and pubertal status (PDS). Model output and BFs are shown in table S6. Alpha, RL rate; Phi, WM Decay; Rho, WM prior weight; Pers, positive learning bias; C, WM capacity; Eta, scaling drift rate; k, relative threshold; A, start point variability.

| **Task performance** | | | | | | | | | |  |
| --- | --- | --- | --- | --- | --- | --- | --- | --- | --- | --- |
| **Predictor** | **Estimate** | **Est.Error** | **l-95% CI** | **u-95% CI** | **OR** | **Rhat** | **p_over_0** | **p_under_0** | **BF_01_** | |
| Intercept | 2.82 | 0.04 | 2.74 | 2.90 | 16.81 | 1.00 | NA | NA | NA | |
| Age | 0.13 | 0.04 | 0.04 | 0.21 | 1.13 | 1.00 | 1.00 | 0.00 | 0.26 | |
| Sex | −0.14 | 0.08 | −0.31 | 0.02 | 0.87 | 1.00 | 0.04 | 0.96 | 2.80 | |
| Delay | −0.06 | 0.01 | −0.09 | −0.04 | 0.94 | 1.00 | 0.00 | 1.00 | 0.00 | |
| Set size | −0.25 | 0.01 | −0.28 | −0.22 | 0.78 | 1.00 | 0.00 | 1.00 | 0.00 | |
| Reward history | 1.74 | 0.02 | 1.70 | 1.78 | 5.72 | 1.00 | 1.00 | 0.00 | 0.00 | |
| Task block | 0.09 | 0.01 | 0.06 | 0.11 | 1.09 | 1.00 | 1.00 | 0.00 | 0.00 | |
| SMFQ sum | −0.02 | 0.06 | −0.13 | 0.10 | 0.98 | 1.00 | 0.40 | 0.60 | 15.67 | |
| GAD-7 sum | 0.00 | 0.06 | −0.11 | 0.12 | 1.00 | 1.00 | 0.52 | 0.48 | 16.38 | |
| Age × Sex | −0.10 | 0.07 | −0.25 | 0.04 | 0.90 | 1.00 | 0.08 | 0.92 | 4.90 | |
| Age × Delay | 0.01 | 0.01 | −0.02 | 0.03 | 1.01 | 1.00 | 0.72 | 0.28 | 63.63 | |
| Age × Set size | −0.01 | 0.01 | −0.03 | 0.02 | 0.99 | 1.00 | 0.35 | 0.65 | 64.83 | |
| Age × Reward history | 0.03 | 0.02 | −0.01 | 0.07 | 1.03 | 1.00 | 0.90 | 0.10 | 21.61 | |
| Age × Task block | 0.01 | 0.01 | −0.02 | 0.03 | 1.01 | 1.00 | 0.70 | 0.30 | 69.46 | |
| Age × SMFQ sum | −0.01 | 0.05 | −0.12 | 0.10 | 0.99 | 1.00 | 0.43 | 0.57 | 18.00 | |
| SMFQ sum × Sex | −0.12 | 0.12 | −0.36 | 0.12 | 0.89 | 1.00 | 0.16 | 0.84 | 4.94 | |
| SMFQ sum × Delay | 0.01 | 0.02 | −0.03 | 0.05 | 1.01 | 1.00 | 0.75 | 0.25 | 39.23 | |
| SMFQ sum × Set size | −0.02 | 0.02 | −0.06 | 0.02 | 0.98 | 1.00 | 0.17 | 0.83 | 29.85 | |
| SMFQ sum × Reward history | −0.04 | 0.03 | −0.10 | 0.02 | 0.96 | 1.00 | 0.12 | 0.88 | 16.02 | |
| SMFQ sum × Task block | −0.06 | 0.02 | −0.09 | −0.02 | 0.94 | 1.00 | 0.00 | 1.00 | 0.57 | |
| Age × GAD-7 sum | −0.04 | 0.05 | −0.14 | 0.07 | 0.96 | 1.00 | 0.24 | 0.76 | 14.35 | |
| GAD-7 sum × Sex | 0.03 | 0.12 | −0.21 | 0.26 | 1.03 | 1.00 | 0.59 | 0.41 | 7.90 | |
| GAD-7 sum × Delay | −0.01 | 0.02 | −0.04 | 0.03 | 0.99 | 1.00 | 0.40 | 0.60 | 48.48 | |
| GAD-7 sum × Set size | 0.00 | 0.02 | −0.04 | 0.04 | 1.00 | 1.00 | 0.46 | 0.54 | 46.61 | |
| GAD-7 sum × Reward history | 0.04 | 0.03 | −0.02 | 0.10 | 1.04 | 1.00 | 0.92 | 0.08 | 11.60 | |
| GAD-7 sum × Task block | 0.04 | 0.02 | 0.00 | 0.07 | 1.04 | 1.00 | 0.97 | 0.03 | 8.15 | |

**Table S2:** Model output from Bayesian mixed-effects logistic regression with trial-level accuracy as dependent variable. All Rhat values were <1.01. Sex was coded as 0 = Female, 1 = Male.

| **PDS Female model** | | | | | | | | |  |
| --- | --- | --- | --- | --- | --- | --- | --- | --- | --- |
| **Predictor** | **Estimate** | **Est.Error** | **l-95% CI** | **u-95% CI** | **Rhat** | **p_over_0** | **p_under_0** | **BF_01_** | |
| Intercept[1] | −3.3953 | 0.2072 | −3.8183 | −3.0127 | 1.0003 | NA | NA | NA | |
| Intercept[2] | −1.2113 | 0.0926 | −1.3960 | −1.0325 | 1.0002 | NA | NA | NA | |
| Intercept[3] | −1.0371 | 0.0886 | −1.2121 | −0.8644 | 1.0004 | NA | NA | NA | |
| Intercept[4] | −0.2145 | 0.0795 | −0.3705 | −0.0602 | 1.0000 | NA | NA | NA | |
| Alpha | 0.0182 | 0.1158 | −0.2106 | 0.2453 | 1.0003 | 0.5637 | 0.4363 | 8.6034 | |
| Phi | −0.0607 | 0.0849 | −0.2281 | 0.1070 | 1.0000 | 0.2396 | 0.7604 | 9.0805 | |
| Rho | 0.1864 | 0.2525 | −0.3098 | 0.6782 | 1.0001 | 0.7693 | 0.2307 | 3.0216 | |
| Pers | 0.0340 | 0.0780 | −0.1165 | 0.1874 | 1.0001 | 0.6688 | 0.3312 | 11.0832 | |
| C | −0.1692 | 0.2736 | −0.7080 | 0.3688 | 1.0002 | 0.2683 | 0.7317 | 2.9331 | |
| Eta | 0.0404 | 0.1146 | −0.1878 | 0.2643 | 1.0004 | 0.6379 | 0.3621 | 8.0431 | |
| k | −0.1955 | 0.0926 | −0.3781 | −0.0138 | 1.0003 | 0.0170 | 0.9830 | 1.2232 | |
| A | −0.3215 | 0.0958 | −0.5071 | −0.1340 | 1.0000 | 0.0006 | 0.9994 | 0.0486 | |

**Table S3:** Model output from Bayesian regression with PDS item response as dependent variable with female participants. All Rhat values were <1.01. Posterior distributions are shown in Fig. S11. Alpha, RL rate; Phi, WM Decay; Rho, WM prior weight; Pers, positive learning bias; C, WM capacity; Eta, scaling drift rate; k, relative threshold; A, start point variability.

| **PDS Male model** | | | | | | | | |
| --- | --- | --- | --- | --- | --- | --- | --- | --- |
| **Predictor** | **Estimate** | **Est.Error** | **l-95% CI** | **u-95% CI** | **Rhat** | **p_over_0** | **p_under_0** | **BF_01_** |
| Intercept[1] | −6.7194 | 0.7696 | −8.3206 | −5.3226 | 1.0003 | NA | NA | NA |
| Intercept[2] | −3.9854 | 0.4787 | −4.9651 | −3.0941 | 1.0003 | NA | NA | NA |
| Intercept[3] | −2.5349 | 0.4060 | −3.3683 | −1.7601 | 1.0000 | NA | NA | NA |
| Intercept[4] | 0.1621 | 0.3576 | −0.5300 | 0.8669 | 1.0000 | NA | NA | NA |
| Alpha | −0.3577 | 0.4325 | −1.2057 | 0.4975 | 1.0006 | 0.2011 | 0.7989 | 1.6324 |
| Phi | 0.2190 | 0.3398 | −0.4535 | 0.8951 | 1.0003 | 0.7407 | 0.2593 | 2.3768 |
| Rho | −0.5310 | 0.5392 | −1.5920 | 0.5262 | 1.0005 | 0.1607 | 0.8393 | 1.0754 |
| Pers | −0.3310 | 0.3530 | −1.0272 | 0.3635 | 1.0002 | 0.1691 | 0.8309 | 1.7769 |
| C | 0.1020 | 0.4615 | −0.8041 | 1.0148 | 1.0004 | 0.5853 | 0.4147 | 2.1097 |
| Eta | −0.1787 | 0.3757 | −0.9175 | 0.5612 | 1.0009 | 0.3127 | 0.6873 | 2.3156 |
| k | 0.4623 | 0.3737 | −0.2652 | 1.1940 | 1.0004 | 0.8926 | 0.1074 | 1.2243 |
| A | −0.2984 | 0.3414 | −0.9698 | 0.3750 | 1.0007 | 0.1881 | 0.8119 | 1.9640 |

**Table S4:** Model output from Bayesian regression with PDS item response as dependent variable with male participants. All Rhat values were <1.01. Posterior distributions are shown in Fig. S11. Alpha, RL rate; Phi, WM Decay; Rho, WM prior weight; Pers, positive learning bias; C, WM capacity; Eta, scaling drift rate; k, relative threshold; A, start point variability.

| **Age model** | | | | | | | | |
| --- | --- | --- | --- | --- | --- | --- | --- | --- |
| **Predictor** | **Estimate** | **Est.Error** | **l-95% CI** | **u-95% CI** | **Rhat** | **p_over_0** | **p_under_0** | **BF_01_** |
| Intercept | 17.740 | 0.199 | 17.349 | 18.135 | 1.000 | 1.000 | 0.000 | NA |
| Sex1 | −0.219 | 0.371 | −0.943 | 0.516 | 1.000 | 0.278 | 0.722 | 2.375 |
| Alpha | 0.121 | 0.274 | −0.422 | 0.658 | 1.000 | 0.676 | 0.324 | 3.290 |
| Phi | −0.007 | 0.210 | −0.417 | 0.409 | 1.000 | 0.488 | 0.512 | 4.864 |
| Rho | 0.154 | 0.447 | −0.731 | 1.014 | 1.000 | 0.638 | 0.362 | 2.106 |
| Pers | −0.005 | 0.199 | −0.398 | 0.389 | 1.000 | 0.490 | 0.510 | 5.216 |
| C | −0.034 | 0.471 | −0.944 | 0.887 | 1.000 | 0.471 | 0.529 | 2.125 |
| Eta | 0.021 | 0.266 | −0.501 | 0.541 | 1.000 | 0.534 | 0.466 | 3.763 |
| k | −0.550 | 0.233 | −1.008 | −0.091 | 1.000 | 0.009 | 0.991 | 0.259 |
| A | −1.131 | 0.245 | −1.612 | −0.647 | 1.000 | 0.000 | 1.000 | 0.000 |
| Sex1:Alpha | −0.336 | 0.464 | −1.252 | 0.563 | 1.000 | 0.237 | 0.763 | 1.696 |
| Sex1:Phi | 0.318 | 0.373 | −0.411 | 1.057 | 1.000 | 0.804 | 0.196 | 1.893 |
| Sex1:Rho | −0.267 | 0.578 | −1.408 | 0.863 | 1.000 | 0.320 | 0.680 | 1.586 |
| Sex1:Pers | −0.090 | 0.377 | −0.828 | 0.649 | 1.000 | 0.407 | 0.593 | 2.657 |
| Sex1:C | −0.121 | 0.547 | −1.187 | 0.960 | 1.000 | 0.409 | 0.591 | 1.784 |
| Sex1:Eta | −0.189 | 0.430 | −1.028 | 0.657 | 1.000 | 0.328 | 0.672 | 2.123 |
| Sex1:k | 0.489 | 0.409 | −0.337 | 1.280 | 1.000 | 0.885 | 0.115 | 1.197 |
| Sex1:A | 0.510 | 0.385 | −0.249 | 1.261 | 1.000 | 0.908 | 0.092 | 1.085 |

**Table S5:** Model output from Bayesian regression with Age as dependent variable. All Rhat values were <1.01. Sex was coded as female = 0, male = 1. Posterior distributions are shown in Fig. 5 and S12. Alpha, RL rate; Phi, WM Decay; Rho, WM prior weight; Pers, positive learning bias; C, WM capacity; Eta, scaling drift rate; k, relative threshold; A, start point variability.

| **GAD-7 model** | | | | | | | | |
| --- | --- | --- | --- | --- | --- | --- | --- | --- |
| **Predictor** | **Estimate** | **Est.Error** | **l-95% CI** | **u-95% CI** | **Rhat** | **p_over_0** | **p_under_0** | **BF_01_** |
| Intercept[1] | −1.376 | 0.178 | −1.722 | −1.027 | 1.000 | 0.000 | 1.000 | 0.000 |
| Intercept[2] | 1.290 | 0.178 | 0.942 | 1.646 | 1.000 | 1.000 | 0.000 | 0.000 |
| Intercept[3] | 2.871 | 0.200 | 2.480 | 3.270 | 1.000 | 1.000 | 0.000 | 0.000 |
| Sex1 | −1.130 | 0.345 | −1.809 | −0.454 | 1.000 | 0.001 | 0.999 | 0.015 |
| Alpha | 0.141 | 0.236 | −0.321 | 0.599 | 1.000 | 0.725 | 0.275 | 3.608 |
| Phi | −0.044 | 0.175 | −0.386 | 0.299 | 1.000 | 0.400 | 0.600 | 5.504 |
| Rho | 0.151 | 0.402 | −0.640 | 0.950 | 1.000 | 0.647 | 0.353 | 2.310 |
| Pers | −0.148 | 0.167 | −0.475 | 0.181 | 1.000 | 0.186 | 0.814 | 4.017 |
| C | 0.206 | 0.425 | −0.628 | 1.043 | 1.001 | 0.690 | 0.310 | 2.063 |
| Eta | −0.090 | 0.230 | −0.547 | 0.365 | 1.000 | 0.348 | 0.652 | 4.170 |
| k | −0.185 | 0.198 | −0.577 | 0.206 | 1.001 | 0.175 | 0.825 | 3.248 |
| A | 0.040 | 0.216 | −0.386 | 0.458 | 1.000 | 0.575 | 0.425 | 4.464 |
| Age_z | 0.070 | 0.197 | −0.317 | 0.462 | 1.000 | 0.640 | 0.360 | 4.727 |
| PDS_mean_z | 0.321 | 0.215 | −0.097 | 0.739 | 1.000 | 0.934 | 0.066 | 1.495 |
| Sex1:Alpha | −0.058 | 0.408 | −0.854 | 0.751 | 1.000 | 0.443 | 0.557 | 2.504 |
| Sex1:Phi | 0.201 | 0.324 | −0.441 | 0.834 | 1.000 | 0.734 | 0.267 | 2.527 |
| Sex1:Rho | 0.552 | 0.540 | −0.516 | 1.622 | 1.000 | 0.848 | 0.152 | 1.082 |
| Sex1:Pers | 0.382 | 0.328 | −0.247 | 1.036 | 1.000 | 0.877 | 0.123 | 1.543 |
| Sex1:C | −0.318 | 0.505 | −1.295 | 0.681 | 1.000 | 0.263 | 0.737 | 1.634 |
| Sex1:Eta | 0.216 | 0.376 | −0.520 | 0.947 | 1.000 | 0.717 | 0.283 | 2.295 |
| Sex1:k | 0.221 | 0.360 | −0.485 | 0.922 | 1.000 | 0.730 | 0.270 | 2.303 |
| Sex1:A | 0.442 | 0.339 | −0.213 | 1.103 | 1.000 | 0.905 | 0.095 | 1.292 |

**Table S6**: Model output from the Bayesian ordinal regression with GAD-7 item response as dependent variable (Fig. 7). Sex was coded as female = 1, male = 0. All posterior distributions for associations are shown in fig. S13. Alpha, RL rate; Phi, WM Decay; Rho, WM prior weight; Pers, positive learning bias; C, WM capacity; Eta, scaling drift rate; k, relative threshold; A, start point variability.

| **SMFQ model** | | | | | | | | |
| --- | --- | --- | --- | --- | --- | --- | --- | --- |
| **Predictor** | **Estimate** | **Est.Error** | **l-95% CI** | **u-95% CI** | **Rhat** | **p_over_0** | **p_under_0** | **BF_01_** |
| Intercept[1] | −0.476 | 0.199 | −0.862 | −0.076 | 1.001 | 0.010 | 0.990 | 0.349 |
| Intercept[2] | 1.929 | 0.205 | 1.537 | 2.343 | 1.001 | 1.000 | 0.000 | 0.000 |
| Sex1 | −0.993 | 0.392 | −1.755 | −0.224 | 1.001 | 0.006 | 0.994 | 0.116 |
| Alpha | 0.317 | 0.267 | −0.205 | 0.837 | 1.000 | 0.882 | 0.118 | 1.923 |
| Phi | −0.193 | 0.205 | −0.596 | 0.205 | 1.002 | 0.170 | 0.830 | 3.242 |
| Rho | 0.224 | 0.439 | −0.632 | 1.081 | 1.001 | 0.694 | 0.306 | 1.989 |
| Pers | 0.064 | 0.195 | −0.319 | 0.447 | 1.000 | 0.629 | 0.371 | 4.822 |
| C | 0.132 | 0.464 | −0.770 | 1.036 | 1.000 | 0.607 | 0.393 | 2.111 |
| Eta | 0.365 | 0.262 | −0.149 | 0.876 | 1.001 | 0.916 | 0.084 | 1.490 |
| k | −0.266 | 0.231 | −0.714 | 0.194 | 1.002 | 0.123 | 0.877 | 2.345 |
| A | 0.240 | 0.254 | −0.255 | 0.734 | 1.002 | 0.828 | 0.172 | 2.518 |
| Age_z | −0.093 | 0.229 | −0.545 | 0.352 | 1.001 | 0.342 | 0.658 | 4.094 |
| PDS_mean_z | 0.409 | 0.244 | −0.067 | 0.886 | 1.001 | 0.956 | 0.044 | 0.988 |
| Sex1:Alpha | 0.140 | 0.467 | −0.765 | 1.061 | 1.001 | 0.616 | 0.384 | 2.019 |
| Sex1:Phi | 0.540 | 0.378 | −0.207 | 1.270 | 1.001 | 0.924 | 0.076 | 0.921 |
| Sex1:Rho | 0.743 | 0.578 | −0.402 | 1.871 | 1.000 | 0.899 | 0.101 | 0.777 |
| Sex1:Pers | −0.003 | 0.380 | −0.746 | 0.745 | 1.001 | 0.499 | 0.501 | 2.689 |
| Sex1:C | −0.207 | 0.543 | −1.251 | 0.861 | 1.000 | 0.348 | 0.652 | 1.716 |
| Sex1:Eta | −0.247 | 0.428 | −1.081 | 0.589 | 1.001 | 0.281 | 0.719 | 2.027 |
| Sex1:k | 0.285 | 0.417 | −0.539 | 1.110 | 1.001 | 0.753 | 0.247 | 1.903 |
| Sex1:A | 0.247 | 0.387 | −0.516 | 1.009 | 1.002 | 0.739 | 0.261 | 2.164 |

**Table S7**: Model output from the Bayesian ordinal regression with SMFQ item response as dependent variable (Fig. 7). Sex was coded as female = 1, male = 0. All posterior distributions for associations are shown in fig. S14. Alpha, RL rate; Phi, WM Decay; Rho, WM prior weight; Pers, positive learning bias; C, WM capacity; Eta, scaling drift rate; k, relative threshold; A, start point variability.

**References**

Collins, A. G. E., & Frank, M. J. (2012). How much of reinforcement learning is working memory, not reinforcement learning? A behavioral, computational, and neurogenetic analysis. *European Journal of Neuroscience*, *35*(7), 1024–1035. https://doi.org/10.1111/j.1460-9568.2011.07980.x

Frank, M. J., Moustafa, A. A., Haughey, H. M., Curran, T., & Hutchison, K. E. (2007). Genetic triple dissociation reveals multiple roles for dopamine in reinforcement learning. *Proceedings of the National Academy of Sciences*, *104*(41), 16311–16316. https://doi.org/10.1073/pnas.0706111104

Hauser, T. U., Iannaccone, R., Walitza, S., Brandeis, D., & Brem, S. (2015). Cognitive flexibility in adolescence: Neural and behavioral mechanisms of reward prediction error processing in adaptive decision making during development. *NeuroImage*, *104*, 347–354. https://doi.org/10.1016/j.neuroimage.2014.09.018

Lefebvre, G., Lebreton, M., Meyniel, F., Bourgeois-Gironde, S., & Palminteri, S. (2017). Behavioural and neural characterization of optimistic reinforcement learning. *Nature Human Behaviour*, *1*(4), 1–9. https://doi.org/10.1038/s41562-017-0067

Master, S. L., Eckstein, M. K., Gotlieb, N., Dahl, R. E., Wilbrecht, L., & Collins, A. G. E. (2020). Disentangling the systems contributing to changes in learning during adolescence. *Developmental Cognitive Neuroscience*, *41*, 100732. https://doi.org/10.1016/j.dcn.2019.100732

McDougle, S. D., & Collins, A. G. E. (2021). Modeling the influence of working memory, reinforcement, and action uncertainty on reaction time and choice during instrumental learning. *Psychonomic Bulletin & Review*, *28*(1), 20–39. https://doi.org/10.3758/s13423-020-01774-z
